# Identification of fatty acid-related subtypes, the establishment of a prognostic signature, and immune infiltration characteristics in lung adenocarcinoma

**DOI:** 10.18632/aging.204725

**Published:** 2023-05-16

**Authors:** Yuzhi Wang, Xiaoxiao Huang, Hong Fan, Yunfei Xu, Zelin Qi, Yi Zhang, Yi Huang

**Affiliations:** 1 Department of Laboratory Medicine, Deyang People’s Hospital, Deyang 618000, Sichuan, People’s Republic of China; 2 Department of Laboratory Medicine, Liuzhou Hospital of Guangzhou Women and Children’s Medical Center, Liuzhou 545000, Guangxi, People’s Republic of China; 3 Guangxi Clinical Research Center for Obstetrics and Gynecology, Liuzhou 545000, Guangxi, People’s Republic of China; 4 Department of Pathology, Shanghai Jianding District Anting Hospital, Shanghai 200000, People’s Republic of China; 5 Department of Laboratory Medicine, Chengdu Women’s and Children’s Central Hospital, Chengdu 610031, Sichuan, People’s Republic of China; 6 Shengli Clinical Medical College of Fujian Medical University, Fujian Medical University, Fuzhou 350001, Fujian, People’s Republic of China; 7 Department of Clinical Laboratory, Fujian Provincial Hospital, Fuzhou 350001, Fujian, People’s Republic of China; 8 Central Laboratory, Center for Experimental Research in Clinical Medicine, Fujian Provincial Hospital, Fuzhou 350001, Fujian, People’s Republic of China; 9 Fujian Provincial Key Laboratory of Critical Care Medicine, Fujian Provincial Key Laboratory of Cardiovascular Disease, Fuzhou 350001, Fujian, People’s Republic of China

**Keywords:** fatty acid, lung adenocarcinoma, prognosis, tumor microenvironment, immunotherapy

## Abstract

Abnormal fatty acid (FA) metabolism can change the inflammatory microenvironment and promote tumor progression and metastasis, however, the potential association between FA-related genes (FARGs) and lung adenocarcinoma (LUAD) is still unclear. In this study, we described the genetic and transcriptomic changes of FARGs in LUAD patients and identified two different FA subtypes, which were significantly correlated with overall survival and tumor microenvironment infiltrating cells in LUAD patients. In addition, the FA score was also constructed through the LASSO Cox to evaluate the FA dysfunction of each patient. Multivariate Cox analysis proved that the FA score was an independent predictor and created the FA score integrated nomogram, which offered a quantitative tool for clinical practice. The performance of the FA score has been substantiated in numerous datasets for its commendable accuracy in estimating overall survival in LUAD patients. The groups with high and low FA scores exhibited different mutation spectrums, copy number variations, enrichment pathways, and immune status. Noteworthy differences between the two groups in terms of immunophenoscore and Tumor Immune Dysfunction and Exclusion were observed, suggesting that the group with a low FA score was more responsive to immunotherapy, and similar results were also confirmed in the immunotherapy cohort. In addition, seven potential chemotherapeutic drugs related to FA score targeting were predicted. Ultimately, we ascertained that the attenuation of KRT6A expression impeded the proliferation, migration, and invasion of LUAD cell lines. In summary, this research offers novel biomarkers to facilitate prognostic forecasting and clinical supervision for individuals afflicted with LUAD.

## INTRODUCTION

Contemporary cancer statistics demonstrate that lung cancer (LC) constitutes one of the most ubiquitous and fatal oncological afflictions [1], with 2.2 million new cases and 1.8 million fatalities globally in 2020. Approximately 85% of lung cancer incidences are classified as non-small cell lung cancer (NSCLC) [2]; of these, lung adenocarcinoma (LUAD) represents the predominant histological subtype, constituting nearly 63% of NSCLC cases [3]. Although the treatment of LUAD has been vastly improved recently, including new immunotherapy, molecular target, and anti-angiogenesis therapy, however, only a small number of patients benefit from them [4, 5]. Even with the most sophisticated therapies and advancements, the 5-year survival rate of patients is still less than 15% due to tumor metastasis [1]. The lack of accurate clinical classification and prognostic evaluation system is the main problem of LUAD at present. Therefore, more sensitive and effective biomarkers are crucial for the diagnosis and prognosis of LUAD.

Cancer cells frequently exhibit reprogrammed metabolic processes, which allow cancer cells to accumulate metabolic intermediates as a source of cell components and lipid metabolism in cells proliferating at an exponential rate will change as well [6–8]. Fatty acids (FA) synthesized by cancer cells in the process of metabolic capacity can be used for membrane and signal molecule biosynthesis. Cell membrane lipids are mainly phospholipids (PL), such as phosphatidylcholine (PC) and phosphatidylethanolamine (PE). Some of these lipids are derived from acetyl coenzyme A, and many contain FA, which can be obtained from exogenous uptake or de novo synthesis. Although most normal human cells prefer exogenous FA uptake, tumors can synthesize FA and usually show a shift towards de novo FA synthesis. Many types of tumors have been proved to rely on fatty acid oxidation (FAO) to provide ATP to maintain cell growth and survival. Recent studies have found that FAO metabolic reprogramming affects tumor metastasis. Wen et al. found that FA released from adipose tissue of colon cancer patients increases FA uptake of colon cancer cells and up-regulates mitochondrial FAO, promoting the growth and metastasis of colon cancer cells [9]. It has been reported that the key enzyme of FAO is abnormally expressed in malignant tumors, especially in ovarian cancer, and the abnormally high expression of CPT1A is closely related to the poor prognosis of ovarian cancer patients [10, 11]. The metastasis of ovarian cancer first involves the abdominal adipose tissue of the omentum. FA in omental adipocytes is hydrolyzed and liberated subsequent to the engagement between neoplastic cells and adipose tissue. Ovarian tumor cells rely on the FA released by these adipocytes to support rapid growth and continuous peritoneal diffusion through FAO [12]. In breast cancer, excretions from malignant mammary cells incite adipocytes to break down and discharge free fatty acids (FFA), which are assimilated and sequestered by the cancerous cells. This process reciprocally stimulates the augmentation of fatty acid oxidation within the neoplastic cells, thereby fostering the metastatic spread of breast cancer cells [13]. The process of neoplastic metastasis encompasses numerous stages. When the tumor develops to a certain stage, it begins to generate blood vessels, and the cancer cells can infiltrate into blood vessels and migrate to other organs for colonization through the blood tract [13, 14]. Cancer cells can also fall off from the primary focus, enter lymphatic vessels and migrate to regional lymph nodes, survive in the lymphatic system, and colonize the target organs after extravasation [15]. FAO is closely related to these steps and a study conducted by Schoor et al. found that FAO supports endothelial cells to generate blood vessels, and abundant blood vessels play an important role in tumor metastasis [15]. Wong et al. confirmed that FAO was up-regulated in lymphatic endothelial cells, and FAO could promote lymphatic endothelial cells to generate lymphatic vessels, which was conducive to lymph node metastasis of cancer cells [16]. Therefore, FA metabolism may become a promising research direction for cancer in the future. However, despite our increasing understanding of this topic, the role of FA metabolism in the prognosis and treatment of LUAD remains unclear.

This investigation aimed to devise a scoring indicator for stratifying LUAD patients based on fatty acid related metabolic genes (FARGs) expression, with the intent of prognostic forecasting and directing therapeutic approaches. Consequently, the FA score can be derived by constructing FA-related models employing the Least Absolute Shrinkage and Selection Operator (LASSO) Cox approach. This score facilitates predictions of patient outcomes, immune infiltration, and immunotherapeutic responsiveness. Our findings revealed potential correlations between FA metabolism and LUAD patient prognoses, the immune microenvironment, and treatment responses.

## MATERIALS AND METHODS

### Data source and preprocessing

**Figure 1:**
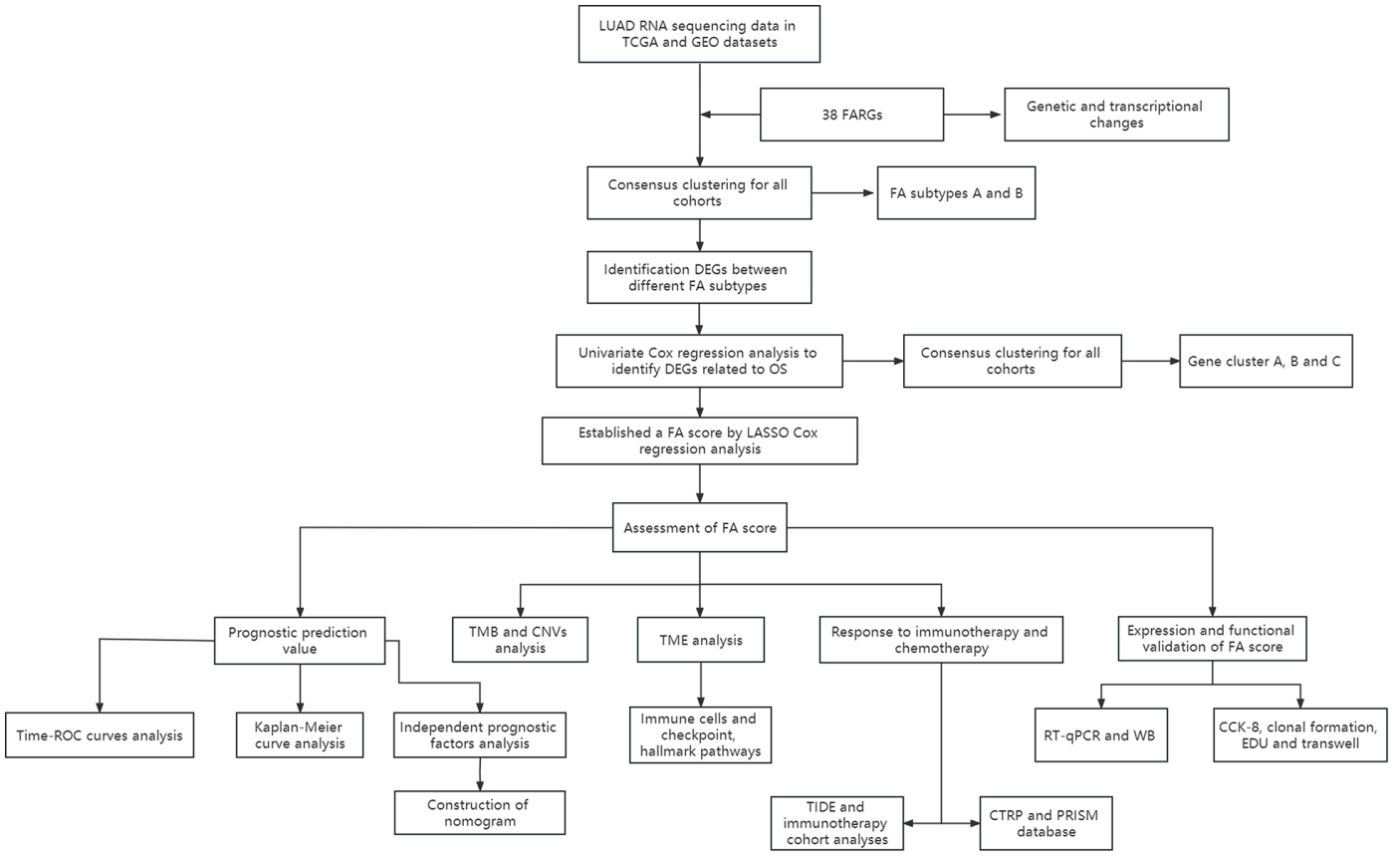
Schematic representation of the present study.

The data of 539 patients diagnosed with LUAD were retrieved from the Cancer Genome Atlas (TCGA) database (https://portal.gdc.cancer.gov/), including RNA sequencing transcriptome (TPM format), mutation, copy number variation (CNV) and clinical data. The microarray data (GSE31210, GSE68465 and GSE72094) of three LUAD patient cohorts were downloaded from the Gene Expression Omnibus (GEO) database (http://www.ncbi.nlm.nih.gov/geo/). Patients with incomplete survival information were excluded from our cohort. The batch effect caused by non-biotechnology deviation among different data sets was reduced by using ‘SVA’ R package’s ‘combat’ algorithm [17]. In total, 503 patients were used from the TCGA database as training sets, and 1066 patients from the GEO database were included in our study as external validation sets. The baseline clinical information of patients was presented in Supplementary Table 1. The procedure for this study is exemplified in Figure 1.

### Unsupervised cluster analysis

Gene sets related to FA metabolic processes were downloaded from the molecular marker database (MsigDB, https://www.GSEA-msigdb.org/GSEa/msigdb/genesets.jsp) as FARGs. We identified 38 overlapping FARGs (Supplementary Table 2) in all datasets and extracted the expression level of FARGs from each case for further analysis. Using unsupervised cluster analysis, different FA subtypes were determined according to 38 FARGs, and all patients were classified. The ‘ConsensusClusterPlus’ R package was employed to perform these analyses and it was repeated 1000 times to ensure the stability of clustering. The optimal number of clusters was determined according to the consistent clustering algorithm.

### Gene set variation analysis (GSVA) and single sample gene set enrichment analysis (ssGSEA)

In order to study the differences in biological processes leading to FA characteristic patterns, the ‘GSVA’ R package was used to perform GSVA [18, 19]. Additionally, GSVA was also performed by downloading ‘c2.cp.kegg.v6.2.symbols’ from MsigDB. Calibration p<0.05 was considered as the statistical significance of the ‘limma’ software package among different subgroups. Simultaneously, the tumor-infiltrating immune cells in each sample were quantified using ssGSEA algorithm of the ‘GSVA’ R package. In addition, the distribution of LUAD patients according to the mRNA expression level of FARGs was shown by using principal component analysis (PCA).

### Determination and annotation of differentially expressed genes (DEGs) in different FA metabolic patterns

‘limma’ package was used to obtain DEGs among patients with different FA subtypes and the significance criteria for selecting DEGs were set as false discovery rate (FDR) <0.05 and | log2 fold change (FC) | ≥ 1. Database for Annotation, Visualization and Integrated Discovery (DAVID, https://david.ncifcrf.gov/) tools were then used to perform Gene Ontology (GO) and Kyoto Encyclopedia of Genes and Genomes (KEGG) enrichment pathway analysis to conclude the potential function of these DEGs. FDR <0.05 was considered to be statistically significant.

### Construction and validation of FA metabolism score

Univariate Cox analysis was used to distinguish DEGs related to prognosis. The generation of gene clusters and evaluation of their stability was done by the unsupervised clustering algorithm. Furthermore, the LASSO method in the ‘survminer’ package was used to deal with them to avoid overfitting and deleting those closely related genes and the minimum penalty item (λ) was selected by utilizing five-fold cross-validation. The FA-related prognostic marker was established for LUAD patients, and the calculation formula for FA score was as follows: FA score= e^sum(UBE2S expression* 0.150928887713532+ HMMR expression* 0.153839775933459+ TMPRSS11E expression* 0.0892095657835886+^ ^CHIT1 expression *-0.0625439224900231 +KRT6A expression* 0.125096336613015)^. Two groups of patients were created following the median FA score: the high FA score group and the low FA score group. The Kaplan-Meier (K-M) survival curve was drawn, and the differences between various FA score groups were evaluated with a log-rank test. The ‘time-dependent receiver operating characteristic (time-ROC)’ package was used to construct the time-ROC curve, which was then validated in three GEO cohorts. The additional prognostic value of the FA score was further confirmed by stratified analysis, and univariate and multivariate cox analyses were both used to identify the independent prognostic indicators of LUAD.

### Construction of nomogram

According to the results of univariate and multivariate cox analysis, the ‘rma’ R package was used to construct a comprehensive nomogram of independent factors to quantitatively evaluate the prognosis of FA score [20]. The nomogram was tested for its accuracy by constructing a calibration curve of 1, 3 and 5 years. The predictive ability of the nomogram was evaluated by using Concordance index (C-index) curve and the time-ROC curve. The nomogram’s prognostic value and FA score were compared according to the C-index [21]. In addition, the net benefit of the nomogram was measured by decision curve analysis (DCA).

### Analysis of genome variation

In order to explore the somatic mutation of FA score, the waterfall diagram was drawn with the ‘maftools’ R package to show the mutation of different FA score groups in LUAD patients. The TMB value reflecting the total mutation variable of each LUAD patient was calculated by non-synonymous mutation, and 38MB was used as the estimated value of exon size [22, 23]. In addition, ‘maftools’ R package was employed for the analysis of the significantly mutated genes and the interaction of gene mutations between high and low FA score groups. In these two analyses, only genes with more than 30 mutations in at least one group were considered. The GISTIC2.0 algorithm was employed to study the changes in somatic copy number between two different FA score groups. The fraction genome altered (FGA), the fraction of genome gained (FGG) and the fraction of the genome lost (FGL) values of each LUAD sample was determined [24]. The gene position on the chromosome was located using ‘RCirco’ R package.

### Analysis of tumor immune characteristics and pathway enrichment of FA score

The evaluation of tumor immune characteristics includes two aspects: (1) The expression level of immune checkpoints [25]; (2) The score and degree of infiltration of infiltrative immunity and stromal cells calculated by Estimation of Stromal and Immune cells in malignant Tumor tissues using Expression data (ESTIMATE) [26] and ssGSEA algorithm [27]. GSEA was employed to enrich potential pathways related to FA score, whereas GSVA was utilized to verify the tumor pathway differences between high and low FA score groups. FDR values <0.05 were considered significant enrichment.

### Prediction of immunotherapy response and chemosensitivity

The clinical response of patients to immune checkpoint inhibitors (ICIs) was predicted using Tumor Immune Dysfunction and Exclusion (TIDE) algorithm [28]. A high TIDE score indicated a worse response to immunotherapy. The calculation process of immunophenoscore (IPS) was described in the previous article [29], where we observed a higher score indicated a better effect of immune checkpoint inhibition treatment. We can obtain IPS from the cancer immune Atlas website (TCIA, https://tcia.at/home). The sensitivity of patients to chemotherapeutic drugs was predicted using CTRP2.0 and PRISM databases, containing the data of area under the curve (AUC) of drug sensitivity, which is used as the standard to measure the drug sensitivity by these two databases. A lower value of the AUC indicates a higher sensitivity to the treatment [30]. Additionally, for the evaluation of the FA score’s potential to predict the response to immunotherapy, an independent anti-PD-L1 immunotherapy cohort (IMvigor210) was included in this study [31].

### Acquisition of tissue samples and cell culture

Fresh cancer and adjacent tissue samples from 30 LUAD patients, who were not treated before surgery, were collected from the Fujian Provincial Hospital. The consent forms were signed by all patients after the specimen extraction, and the ethical approval of the Fujian Provincial Hospital (Ethics Approval Number K2022-05-019) was taken for our research plan. Each specimen was placed in a centrifuge tube containing RNA preservation solution and stored at -80° C. All cell lines were bought from the cell bank of the Chinese Academy of Sciences. BEAS-2B was cultured in high sugar DMEM (Sigma-Aldrich, USA) containing 10% FBS (GIBCO, USA). RPMI-1640 medium (Sigma-Aldrich, USA), containing FBS was used to culture A549, HCC827, and BEAS-2B cells in a humid environment containing with 5% CO2 at 37° C.

### Extraction of total RNA and analysis of real-time quantitative polymerase chain reaction (RT-qPCR)

The Trizol reagent (Invitrogen, USA) was used for total RNA extraction from tissues and cell lines following the manufacturer’s instructions. cDNA synthesis was carried out by RNA reverse transcription using PrimeScript RT kit (Promega Corporation, Madison, WI, USA). RT-qPCR (Roche, Germany) was performed on the Roche LightCycler480 II system according to the instructions of Promega SYBR-Green PCR Master Mix (Promega Corporation, Madison, WI, USA). The determination was carried out on a 96-wells plate, and each sample had three duplicate holes. GAPDH was used as the internal reference gene, and the relative expression level of mRNA was calculated using 2^-ΔΔCT^ method. The primer sequence of RT-qPCR is shown in Supplementary Table 3.

### Western blot analysis

The Western blot analysis was conducted following the established protocol [32]. Tissues and cells underwent homogenization in radioimmunoprecipitation assay buffer (RIPA, Solarbio, China, R0010), supplemented with 1% protease inhibitor cocktail, and the supernatant concentration was ascertained utilizing the bicinchoninic acid assay. Target proteins were loaded onto a sodium dodecyl sulfate-polyacrylamide gel, followed by protein transfer to a polyvinylidene fluoride membrane. The membranes were then incubated overnight at 4° C with the following primary antibodies: KRT6A (ABclonal, China, 1:1000), TMPRSS11E (Thermo Fisher Scientific, USA, 1:1000), HMMR (ABclonal, China, 1:1000), UBE2S (ABclonal, China, 1:1000) CHIT1 (ABclonal, China, 1:1000) and GAPDH (ABclonal, China, 1:1000) antibodies after a 1.5 hour blocking period. Upon washing with Tris-Buffered Saline with EDTA and Tween 20, the membranes were exposed to horseradish peroxidase-conjugated secondary antibodies for 1 hour at ambient temperature. The blots were developed using an enhanced chemiluminescence substrate (ECL reagents) and subsequently analyzed by Image Lab image analysis software.

### Cell transfection

The HanBio Company (Fuzhou, Fujian, China) provided KRT6A small interfering RNAs (siRNA-KRT6A) and non-target small interfering RNA (siRNA-control). Two siRNA and si-control sequences are presented in Supplementary Table 4. Following the manufacturer’s instructions, transfection was carried out using HanBio RNA-specific transfection reagent (Invitrogen, Carlsbad, CA, USA) in Opti-MEM medium (Gibco, Rockville, MD, USA). mRNA expression levels after 48 hours and protein expression levels after 72 hours were carried out to assess the efficiency of cell transfection. After stable transcription, the cells were collected for the other cell-related experiments.

### CCK8 (cell counting kit-8) assay

The cell proliferation ability after transfection was studied by CCK8. Briefly, the KRT6A-knockdown cells in the logarithmic phase of growth were seeded into 96-well plates with 2000 cells per well. After the cells adhered, 10μl CCK8 reagent (Cellcook, Guangzhou, China) was added to the into wells and OD (optical density) was measured after incubation at 37° C and 5% CO2 for 2 hours. The detection time was set as 0, 24, 48 and 72 hours respectively. The absorbance at 450nm for each well was read using a microplate reader.

### Clone formation assay

The transfected cell suspension was seeded in a 6-well plate at 1000 cells/well. Cells in 6-well plates were then grown in a complete medium. The cells were cultured for 10 days, the cells were fixed with 4% paraformaldehyde for 30 minutes and stained with 0.1% crystal violet for 30 minutes. The formed cell colonies were observed and photographed with a microscope. The number of colonies formed was counted by ImageJ.

### 5-Ethynyl-20-deoxyuridine (EdU) assay

Employing an EdU Kit (Beyotime, China, C0071s), the EdU assay was conducted. Approximately 60,000 cells per well were distributed in 24-well plates and subsequently cultured with EdU reagent at a 1:1000 dilution for 2 hours on the subsequent day. The cells were then fixed utilizing 4% paraformaldehyde and stained with fluorescent dye and Hoechst. Post-staining, the cells were imaged and enumerated under a fluorescence microscope. The EdU-positive cell index was ascertained by calculating the proportion of positive cells relative to the total cell count.

### Transwell assay

To determine the migratory capacity of cells, 100μl of the cell suspension (50000 cells) with serum-free medium were seeded into the upper chamber of a transwell plate. Next, 500μl complete 15% FBS-containing medium was added to the lower compartments. After incubation for 24 hours, the cells were fixed with 4% paraformaldehyde and stained with 0.1% crystal violet for 30 minutes respectively. Then a cotton swab was used on the cells in the upper chambers. Eventually, the cells on the lower surface of the membrane were observed and photographed by the microscope, and the results were measured and recorded using ImageJ. For the invasion assay, the upper chamber was coated with 100μl of 10% Matrigel. The rest of the procedure was the same as migration assay.

### Statistical analysis

R software (version 4.2.1), GraphPad Prism (version 9.0) and SangerBox web tool (http://sangerbox.com/) were used to perform all the statistical analyses. Some standard tests which included Student’s T test, Wilcoxon rank sum test, chi-square test, and Fisher exact test, were employed to determine the differences in variables among different groups. The p-value for multivariate multiple comparisons underwent adjustment employing the Benjamin-Hochberg method, and all tests utilized two-tailed p-values [33]. Moreover, the statistical significance was set to p <0.05.

## RESULTS

### Genetic and transcriptional changes of FARGs in LUAD

**Figure 2:**
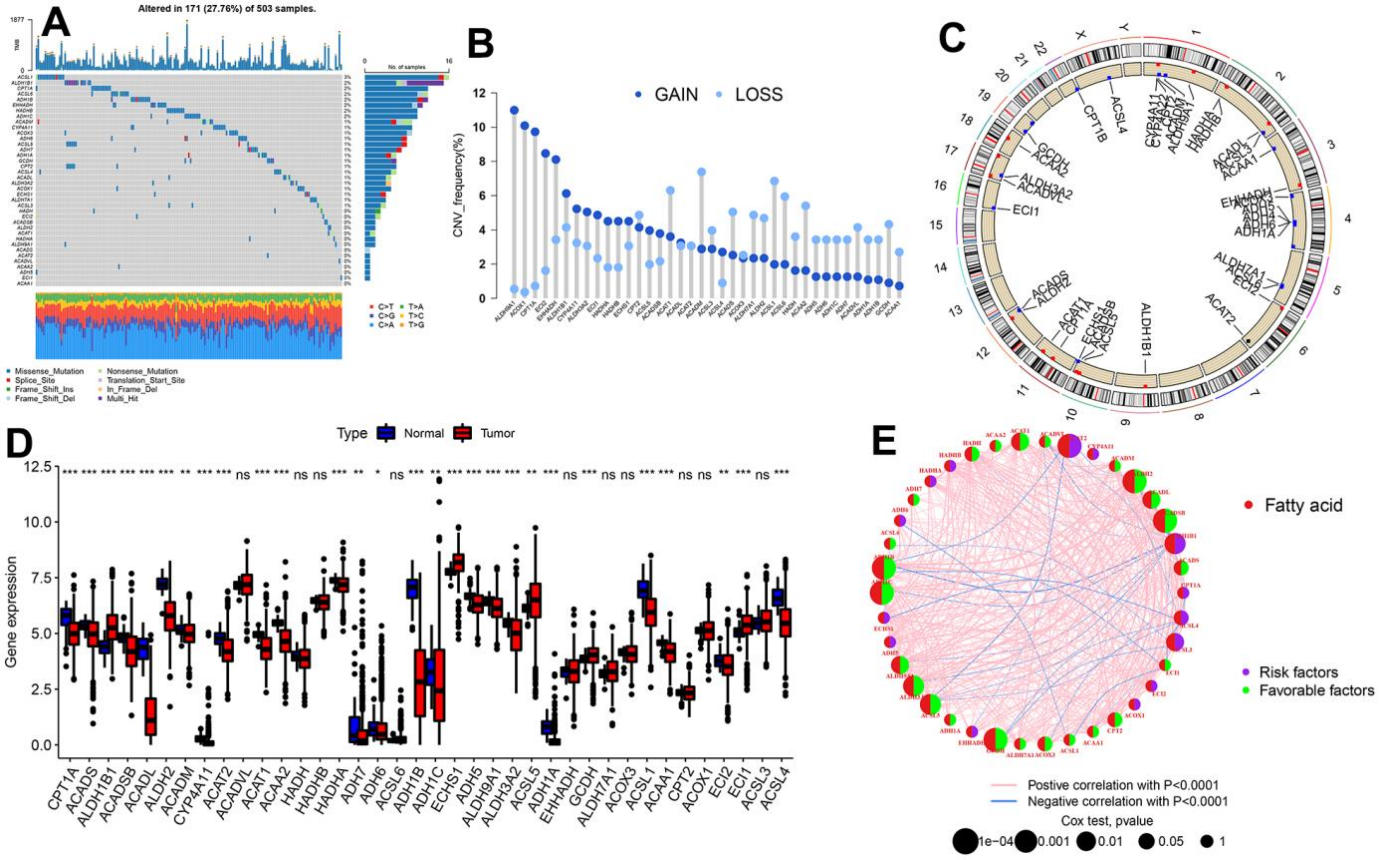
**Genetic and transcriptional alterations of FARGs in TCGA-LUAD patients.** (**A**) Mutation frequencies of 38 FARGs. (**B**) CNV alteration frequency of 38 FARGs. (**C**) Location of the CNV alteration of 38 FARGs on chromosomes. (**D**) Expression of 38 FARGs between normal and tumor tissues. (**E**) The interactions among FARGs in LUAD are visually represented in the diagram. The interconnecting lines between FARGs signify their interdependence, with the line width representing the strength of the correlation between FARGs. Negative correlations are illustrated in green, while positive correlations are denoted in pink. ns, not significant, *P < 0.05, **P < 0.01, ***P < 0.001, ****P < 0.0001.

A total of 38 FARGs were enrolled in this study and the incidence of somatic mutations analysis in these 38 FARGs revealed that the overall frequency of mutation was relatively low in the LUAD cohort (Figure 2A). Only 171 of 503 patients had mutations (Figure 2A), among which ACSL1 had the highest mutation frequency (3%), while 14 FARGs had no mutation. Furthermore, the somatic CNV in these FARGs was studied and it was found that CNV was common in all 38 FARGs (Figure 2B). ALDH9A1 and ACOX1 exhibited a significant increase in CNV, whereas ACADM displayed a decrease in CNV. The location of CNV changes in FARGs on their respective chromosomes is illustrated in Figure 2C. Additionally, the level of expression of all FARGs in tumor and non-tumor tissues were statistically different (Figure 2D). In univariate Cox analysis, 18 FARGs related to prognosis were detected. Figure 2E illustrates the network of FARGs interactions, prognostic values, and regulator connections in patients with LUAD.

### Identification of FA subtypes

**Figure 3:**
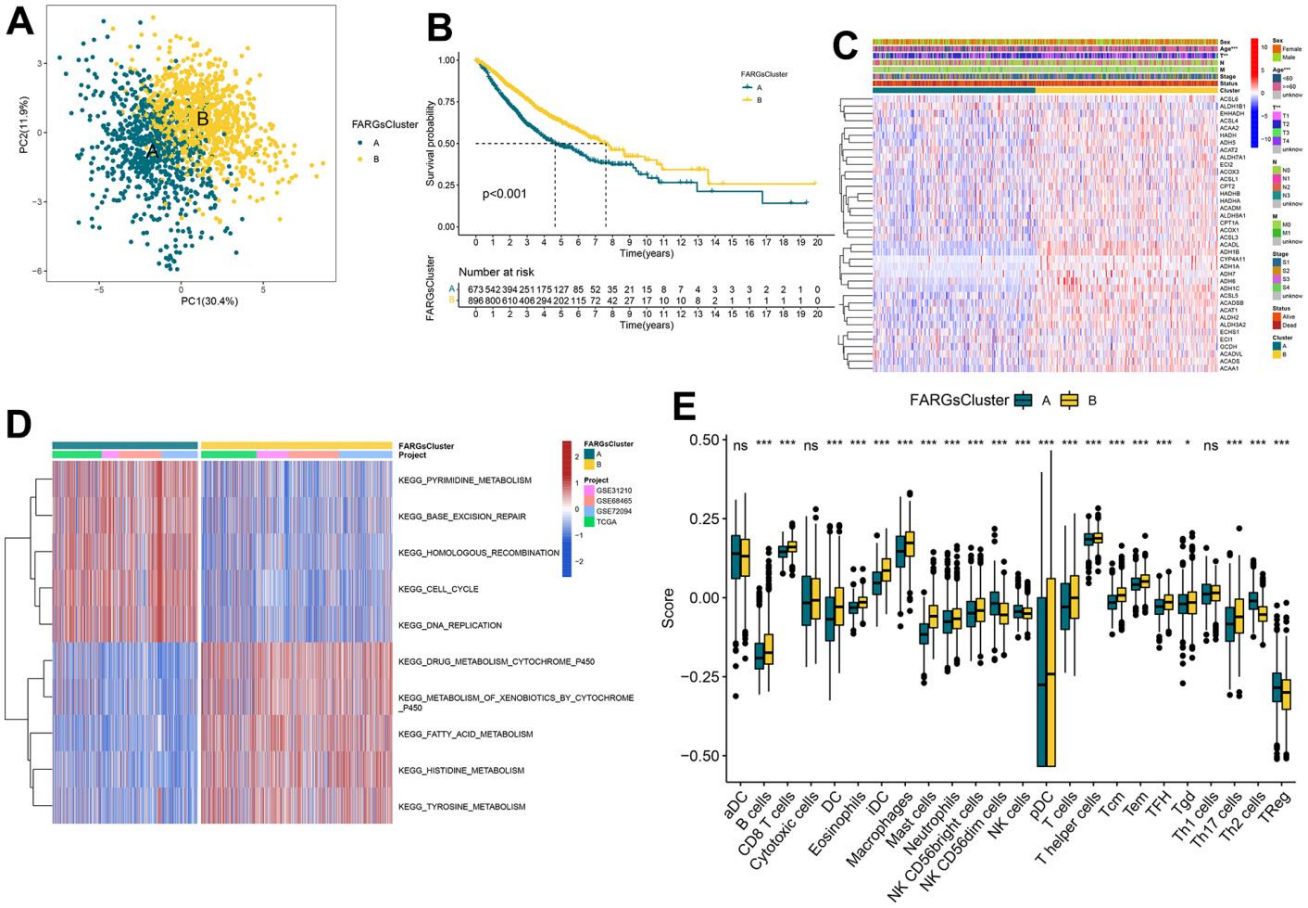
**Consistent clustering for FARGs clusters, biological processes, and characteristics of immune infiltration cells of each cluster.** (**A**) PCA plot based on the FARGs. (**B**) Kaplan–Meier survival curves for the different FARGs clusters. (**C**) Heatmap for different clinicopathologic features and expression levels of FARGs between two clusters. (**D**) GSVA of biological pathways between two clusters. (**E**) Different expressions of immune infiltration cells in each FARGs cluster. ns, not significant, *P < 0.05, **P < 0.01, ***P < 0.001, ****P < 0.0001.

A total of 1569 samples from four independent LUAD cohorts were retained with complete survival information. Through unsupervised clustering of 38 FARGs expressions, the whole cohort was divided into two subtypes: subtype A and B (Supplementary Figure 1 and Supplementary Table 5). Through PCA analysis, significant differences in FARGs transcriptional profiles were revealed between the two subtypes (Figure 3A). Survival analysis revealed that patients with subtype A had a significantly smaller OS than those with subtype B (Figure 3B). Moreover, the comparison of the clinical characteristics of different subtypes of LUAD indicated significant differences in the FARGs expression and clinical pathological characteristics, and the subtype of patients was related to age and T stage (Figure 3C). The KEGG enrichment pathway and the immune cell composition of tumor microenvironment (TME) between the two subtypes were analyzed to understand the association between intrinsic biological traits and various clinical phenotypes. GSVA revealed that subtype A was enriched in the cell process, while subtype B was mainly related to the metabolism of various substances (Figure 3D and Supplementary Table 6). According to the results of the ssGSEA analysis, subtype B had considerably higher levels of infiltration of B cells, DC cells, eosinophils, T cells, and Th cells than subtype A, while subtype A had significantly higher levels of infiltration of NK cells, Treg cells, and Th2 cells than subtype B (Figure 3E and Supplementary Table 7).

### Gene cluster identification based on DEGs

**Figure 4:**
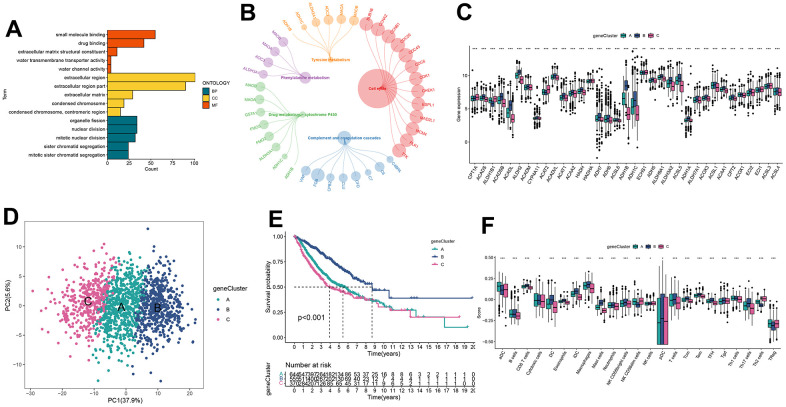
**Identification of gene clusters based on DEGs.** (**A**, **B**) GO and KEGG enrichment analyses of DEGs among two FARGs clusters. (**C**) Expression of FARGs between gene clusters A, B and C. (**D**) PCA plot based on the DEGs. (**E**) Kaplan–Meier curves for the different gene clusters. (**F**) Different expressions of immune infiltration cells in each gene cluster. ns, not significant, *P < 0.05, **P < 0.01, ***P < 0.001, ****P < 0.0001.

To investigate the potential biological behavior inherent in each FA pattern, we identified 222 DEGs related to FARGs subtypes. Subsequently, we conducted a meticulous functional enrichment analysis to gain a deeper understanding of these DEGs (Supplementary Table 8). These DEGs were then enriched and analyzed. The FARGs were significantly enriched in the process of metabolism and cell cycle (Figure 4A, 4B and Supplementary Table 9), indicating that FA plays a significant role in cell cycle and metabolism. The Cox regression analysis was then used to detect the relationship between these FARGs and OS in LUAD patients, and 197 FARGs with strong prognostic values were identified to help with further analysis of the core characteristics of FA (Supplementary Table 10). To verify this conclusion, we undertook an additional unsupervised clustering step on a set of 197 FARGs, which resulted in the identification of three distinct gene clusters for FARGs (Supplementary Figure 2 and Supplementary Table 11). Obviously, significant differences in the mRNA expression of FARGs were observed between gene clusters in the metadata set (Figure 4C). The PCA map displays the relative distances of the three gene clusters using current prognostic DEGs (Figure 4D). Similar to FARGs subtyping, gene clusters can also be utilized to differentiate immune-infiltrating cells and OS in all patients. (Figure 4E, 4F).

### Construction and validation of prognostic FARGs score

**Figure 5:**
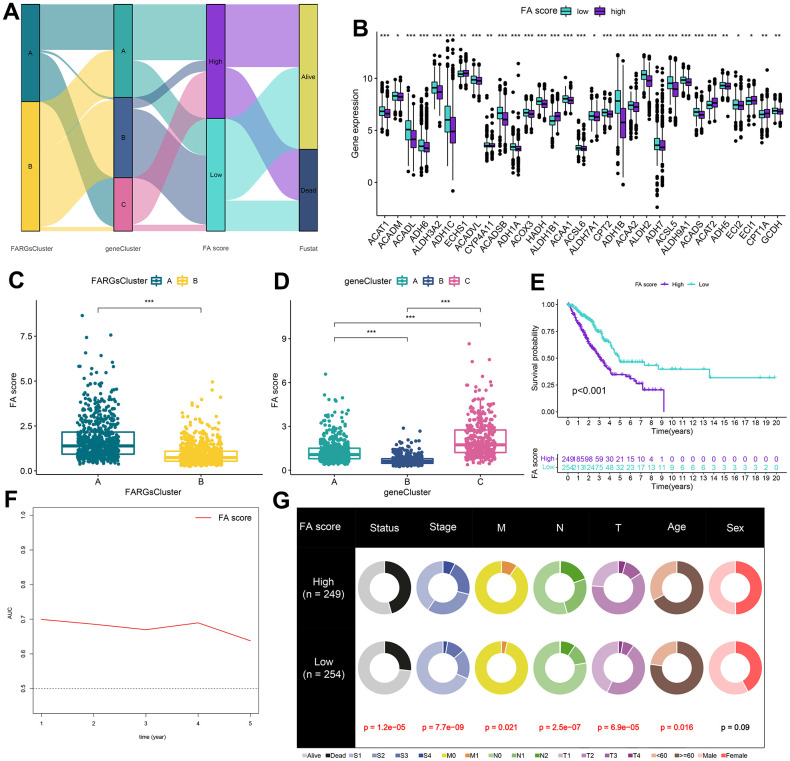
**Construction of FA score.** (**A**) Alluvial diagram of clusters distributions in groups with different FA score and survival outcomes. (**B**) Expression of FARGs between high and low FA score groups. (**C**) Differences in FA score between FARGs clusters. (**D**) Differences in FA score between gene clusters. (**E**) Kaplan–Meier curves for patients with high and low FA score in the TCGA-LUAD cohort. (**F**) Time-dependent receiver operating characteristic curve of FA score for predicting the prognosis of the LUAD patients in the TCGA-LUAD. (**G**) The circular pie chart for the proportion difference of clinical indices. ns, not significant, *P < 0.05, **P < 0.01, ***P < 0.001, ****P < 0.0001.

In the establishment of a model for quantification of each patient, five of the 197 prognostic DEGs were maintained by applying multivariate Cox regression analysis and the LASSO regression model. These were used to establish FARGs scores, named ‘FA score’ (Supplementary Figure 3 and Supplementary Table 12). Furthermore, the value of the FA score was determined by the prediction of the prognosis of patients. The patients were divided into two groups: the high FA score group and the low FA score group according to the median score. Figure 5A illustrates the distribution of patients in two FA subtypes, three gene phenotypes and two FA score groups. There were differences detected in relation to expressions of the great majority of FARGs in the high and low FA score groups (Figure 5B). Besides, significant differences in FA score among FA clusters and gene clusters were identified (Figure 5C, 5D). We observed that the low FA score group had a significant survival advantage over the group with a high FA score (Figure 5E). To showcase the comprehensive significance of the FA score, its validation across supplementary cohorts produced consistent results (Supplementary Figure 4A–4C and Supplementary Table 12). Additionally, with the use of time-ROC analysis, it was further verified that the FA score is a good indicator for predicting the prognosis of LUAD patients (Figure 5F and Supplementary Figure 4D–4F). In order to test the good applicability of the FA score, a stratified analysis of LUAD cancer patients was performed based on clinical and pathological data. The comparison between patients with high and low FA scores revealed that individuals with high FA scores had a shorter OS period at most levels including age over 60 years (Supplementary Figure 5). However, it may be because of the small sample size, and no statistical difference in OS of M1 patients of the high and low FA score groups. The correlation analysis between FA score and several clinical features showed that the survival status, M, N, T, age and stage of LUAD patients were notably associated with FA score (Figure 5G) and these findings indicate that FA score is a highly reliable marker. As a result, the FA score was included in Cox regression analysis as an effective index and clinical characteristics, and we observed that FA score, N, and T are independent variables that impact the prognosis of LUAD patients (Table 1).

**Table 1:** Univariate and multivariate Cox analysis of the clinicopathological features and FA score with OS.

**Characteristics**	**Univariate Cox**		**Multivariate Cox**	
	**HR(95%CI)**	***P* value**	**HR(95%CI)**	***P* value**
Stage	1.977(1.586-2.463)	**< 0.001**	1.256(0.883-1.786)	0.205
M	1.727(1.18-2.527)	**0.005**	1.184(0.75-1.87)	0.469
N	1.942(1.575-2.394)	**< 0.001**	1.584(1.193-2.104)	**0.001**
T	1.816(1.386-2.38)	**< 0.001**	1.495(1.067-2.094)	**0.02**
Age	1.038(0.822-1.31)	0.754		
Sex	1.041(0.847-1.28)	0.7		
FA score	0.579(0.467-0.717)	**< 0.001**	0.695(0.535-0.903)	**0.006**

Significant value is given in bold.

### Construction of nomogram for predicting the survival rate

**Figure 6:**
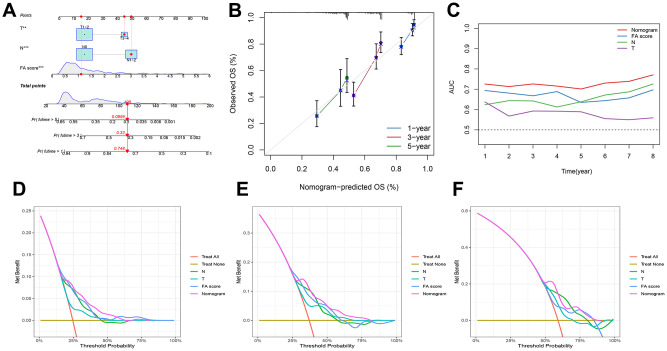
**Development of a nomogram by integrating the FA score and clinicopathological characters in TCGA-LUAD cohort.** (**A**) Nomogram for predicting the 1-, 3-, 5-years OS. (**B**) Calibration curve of the nomogram for predicting the 1-, 3-, and 5-years OS. (**C**) ROC curve for predicting the different years’ OS. (**D**–**F**) Decision curves showing the comparison of net benefits of the nomogram, N, T and FA score for 1-, 3-, and 5-years OS.

Considering the inconvenience of using FA score to predict OS in LUAD patients clinically, a nomogram was established featuring the FA score and clinicopathological parameters to predict the OS rate of patients (Figure 6A). Predictive factors included independent factors N, T, and FA score, and the calibration chart illustrated that the proposed nomogram had a similar performance in the TCGA queue compared with the ideal model (Figure 6B). The time-ROC and C-index curves both suggested that the nomogram had the best prediction effect (Figure 6C, 6D). In addition, DCA disclosed that the nomogram garnered greater net advantages in predicting 1, 3, and 5-year prognostic outcomes compared to univariate analysis (Figure 6E, 6F). More importantly, these results were validated in the independent cohort GSE68465 (Supplementary Figure 6).

### Correlation between genomic changes and FA score

**Figure 7:**
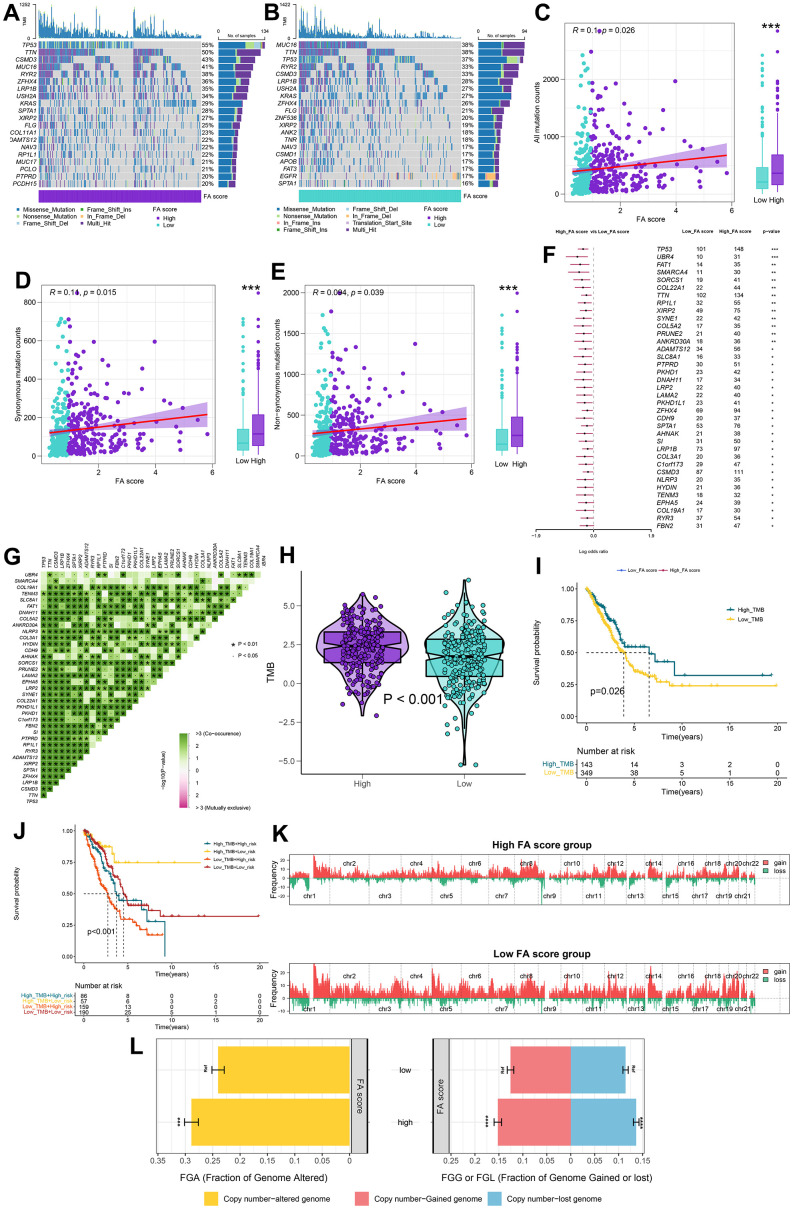
**Integrated comparisons of somatic mutation and CNVs between FA score groups in the TCGA cohort.** (**A**, **B**) Waterfall plots showing the mutation information of the top 20 genes with the highest mutation frequency in high and low FA score groups. (**C**–**E**) Association between all mutation counts, synonymous mutation counts, nonsynonymous mutation counts, and FA score and their distribution in the low and high FA score groups. (**F**) Differentially mutated genes between high and low FA score groups are displayed as a forest plot. (**G**) Interaction effect of genes mutating differentially in patients in the low and the high FA score groups. (**H**) Distribution of TMB in the low and the high FA score groups. (**I**) Kaplan–Meier curves for the OS of the high-TMB and low-TMB groups. (**J**) Kaplan–Meier curves for patients stratified by both TMB and FA score. (**K**) Gene fragments profiles with amplification (red) and deletion (green) among the two groups. (**L**) Comparison of the fraction of the genome altered, lost, and gained between the two groups. ns, not significant, *P < 0.05, **P < 0.01, ***P < 0.001, ****P < 0.0001.

The distribution of somatic variation in LUAD driver genes was evaluated across groups with high and low FA scores. The top 20 driver genes with the highest frequency of change were analyzed and evaluated using the ‘maftools’ tool. (Figure 7A, 7B). These findings provide a new direction for studying the composition of tumor ICIs and the mechanism of gene mutation in immune checkpoint blockade (ICB) treatment. When comparing the mutation frequency between samples from high and low FA score groups, more somatic mutations, including non-synonymous mutations and synonymous mutations, were observed in the high FA score group (Figure 7C–7E). The TCGA cohort mutation annotation file analysis revealed a substantial difference in the mutation frequency of 37 genes, including TP53 and UBR4, and between the two FA score groups, a significant co-occurrence of mutations in these genes was observed (Figure 7F, 7G). Additionally, the patients’ TMB was considerably higher in the high FA score group than that in the low FA score group (Figure 7H and Supplementary Table 13). According to the optimal cut-off value of TMB, patients were divided into two groups. K-M survival analysis revealed that the patients in the high TMB group had a substantially ideal OS rate (Figure 7I). Subsequently, the TMB score and FA score were combined to conduct a stratified survival analysis, which showed that the high and low TMB subgroups classified in accordance with the FA score displayed significant survival differences (Figure 7J). Further analysis was performed of the GISTIC score and copy number gain/loss frequency of high and low FA score groups and the results illustrated that the frequency of copy number increase/decrease in the high FA score group was greater than that in low FA score group (Figure 7K). Furthermore, the differences in FGA, FGG, and FGL between subtypes were evaluated and we observed greater values of the FGA, FGG and FGL in the high FA score group than those in the low FA score group (Figure 7L and Supplementary Table 14). This result indicated that the increase in copy number/loss frequency might be a factor leading to the higher FA score in LUAD patients.

### Evaluation of TME between high FA score and low FA score patients

**Figure 8:**
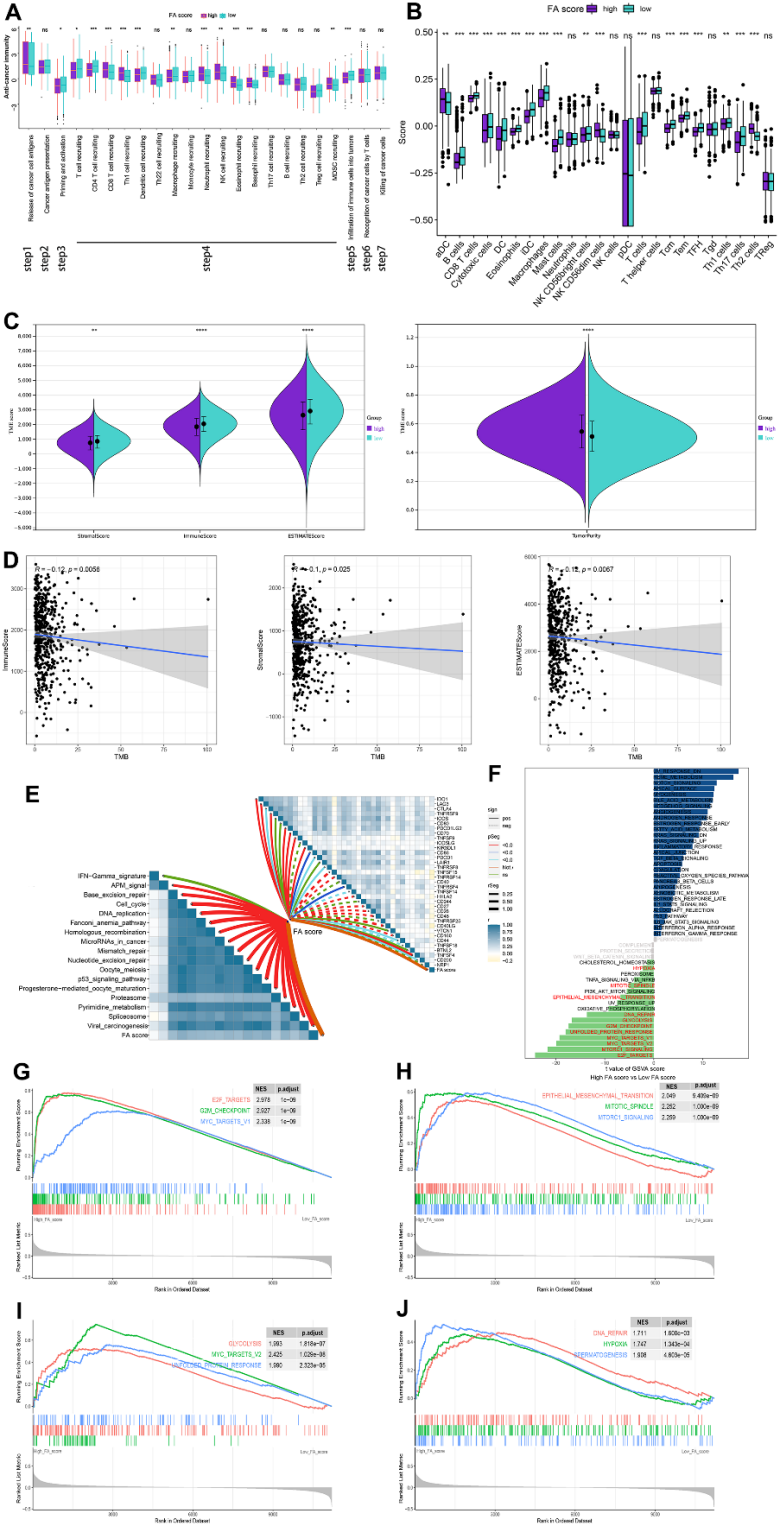
**The relationship between immune characters of TME and FA score in the whole cohort.** (**A**) Differences in activities of the cancer immunity cycles between high and low FA score groups. (**B**) Different expressions of immune infiltration cells in each FA score group. (**C**) Violin plots for the immune score, stromal score, ESTIMATE score, and tumor purity in the low and high FA score groups. (**D**) Spearman correlation analysis between TMB and immune score, stromal score, and ESTIMATE score. (**E**) The correlations between FA score, immunotherapy-predicted pathways and immune checkpoints. (**F**) The difference in the hallmark gene sets between FA score groups. (**G**–**J**) The GSEA results for the 11 overlapping upregulated hallmark pathways in terms of the high FA score groups. ns, not significant, *P < 0.05, **P < 0.01, ***P < 0.001, ****P < 0.0001.

The effects of immunotherapy and its consequences on the cancer cells are determined by the state of TME. The correlation between FA score and cancer immune cycle activity was analyzed and the activity of some anti-cancer immune responses, such as Release of cancer cell antigens, Priming and activation, CD8 T cell recruiting, Neutrophil recruiting and MDSC recruiting, was observed to be substantially different in the high and low FA score groups (Figure 8A and Supplementary Table 15). Moreover, the association between FA score and immune cell abundance was evaluated using ssGSEA algorithm. The scatter diagram illustrates the majority of immune cells were vastly infiltrated in the low FA score group (Figure 8B). A low FA score was closely related to a high immune score, stromal score and ESTIMATE score, while a high FA score was related to high tumor purity (Figure 8C and Supplementary Table 16). Additionally, TME score exhibits a negative correlation with TMB (Figure 8D). The FA score was found to be positively correlated with the expression of many immunological checkpoints as well as the enrichment score of gene features relevant to immunotherapy response (Figure 8E and Supplementary Tables 17, 18). GSVA was conducted in the high FA score and low FA score groups to investigate the cancer marker pathways related to FA score. Compared with the low FA score group, the 17 landmark pathways in the high FA score group were substantially increased (Figure 8F and Supplementary Table 19). Moreover, GSEA corroborated that 11 oncogenic pathways were upregulated in the high FA score group, the majority of which were linked to the well-established carcinogenic pathways (Figure 8G–8J and Supplementary Table 20).

### Application of FA score in predicting chemotherapy and immunotherapy

**Figure 9:**
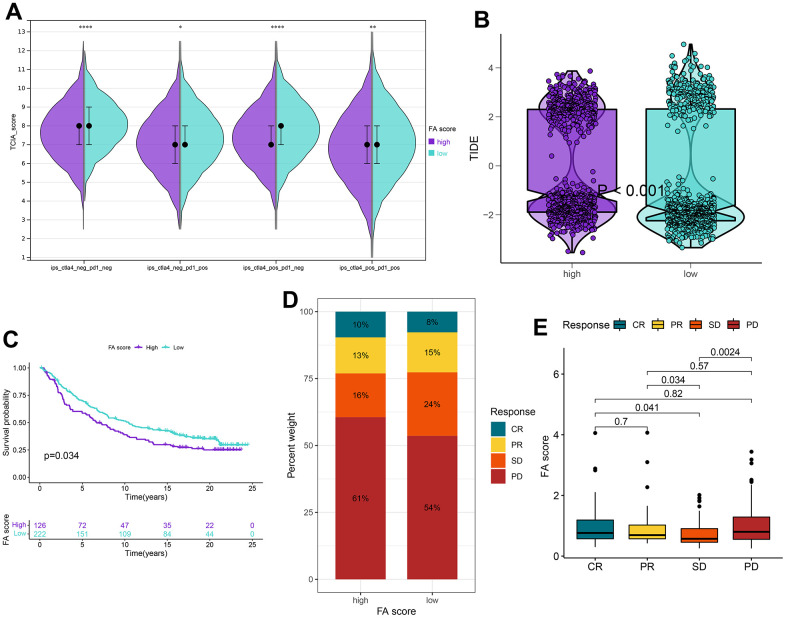
**Application of the FA score for immunotherapy prediction in the high and low FA score groups in the TCGA-LUAD cohort.** (**A**) IPS is used to predict the responsiveness to CTLA-4 and PD-1. (**B**) Distribution of TIDE scores in the whole cohort. (**C**) Kaplan–Meier curves for patients with high and low FA score in the IMvigor210 cohort. (**D**) The rate of response to immunotherapy for patients with high and low FA score in the IMvigor210 cohort. (**E**) The distribution of FA score in different patient statuses in the IMvigor210 cohort. ns, not significant, *P < 0.05, **P < 0.01, ***P < 0.001, ****P < 0.0001.

IPS files downloaded from TCIA were utilized to determine whether FA score can predict the response of LUAD patients to immunotherapy. An elevated IPS value was discerned in the low FA score cohort, signifying an enhanced immunotherapeutic response among the patients (Figure 9A and Supplementary Table 21). The TIDE algorithm was also used to predict the immunotherapeutic effect of ICB which suggested that the TIDE score of the high FA score group was higher, implying that patients with high FA score may display a poor immunotherapy response (Figure 9B and Supplementary Table 22). An immunotherapy cohort (IMvigor210) was introduced to further investigate if an FA score can predict response to immunotherapy. A better survival rate was observed in patients with low FA scores, and they showed a higher objective remission rate than those with high FA score (Figure 9C–9E). While immunotherapy remains a preeminent modality for cancer management, chemotherapy has historically served as a crucial postoperative intervention. Therefore, it is necessary to forecast potential LUAD therapeutic alternatives with the CTRP and PRISM repositories. According to the results, drugs such as leptomycin B and paclitaxel were predicted to be better options for patients with high FA scores. Moreover, it was observed that out of the seven chemotherapy drugs, the value of AUC of gemcitabine was the lowest, which means that LUAD patients may have good therapeutic sensitivity to gemcitabine (Supplementary Figure 7).

### The expression level of genes in FA score in LUAD cell lines and tissues

**Figure 10:**
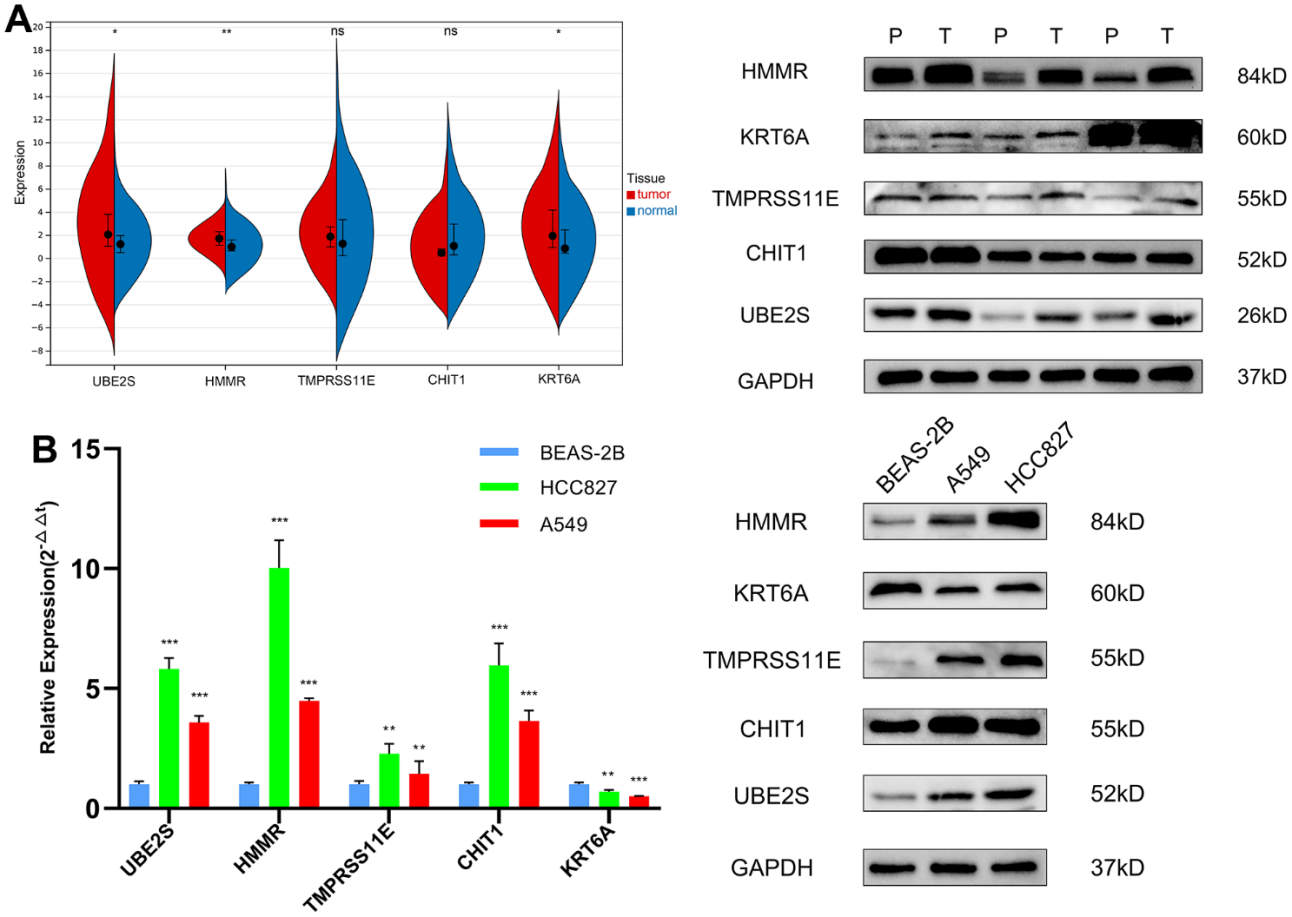
**The expression validation of the genes in the FA score by RT-qPCR and WB.** (**A**) Expression levels of 5 genes in LUAD and matched paracancerous tissues. (**B**) Expression levels of 5 genes in the normal lung epithelial cell line BEAS-2B and two LUAD cell lines (HCC827, A549). ns, not significant, *P < 0.05, **P < 0.01, ***P < 0.001, ****P < 0.0001. T, tumor tissues; P, paracancerous tissues.

UBE2S, HMMR, TMPRSS11E and KRT6A were found to be up-regulated in tumor tissues relative to surrounding non-cancerous and normal tissues in the screening cohort (Supplementary Figure 8). The RT-qPCR and WB for LUAD cell lines and patient tissues were used to validate the expression levels of 4 genes in LUAD. The expression of UBE2S, HMMR and KRT6A expression in tumor tissues was substantially higher than that in adjacent non-cancerous tissues (Figure 10A). For cell lines, the expression of UBE2S, HMMR, TMPRSS11E and CHIT1 was upregulated, whereas KRT6A was downregulated in LUAD cell lines compared with cell line BEAS-2B (Figure 10B).

### Knockdown of KRT6A inhibits LUAD cell proliferation, migration and invasion

**Figure 11:**
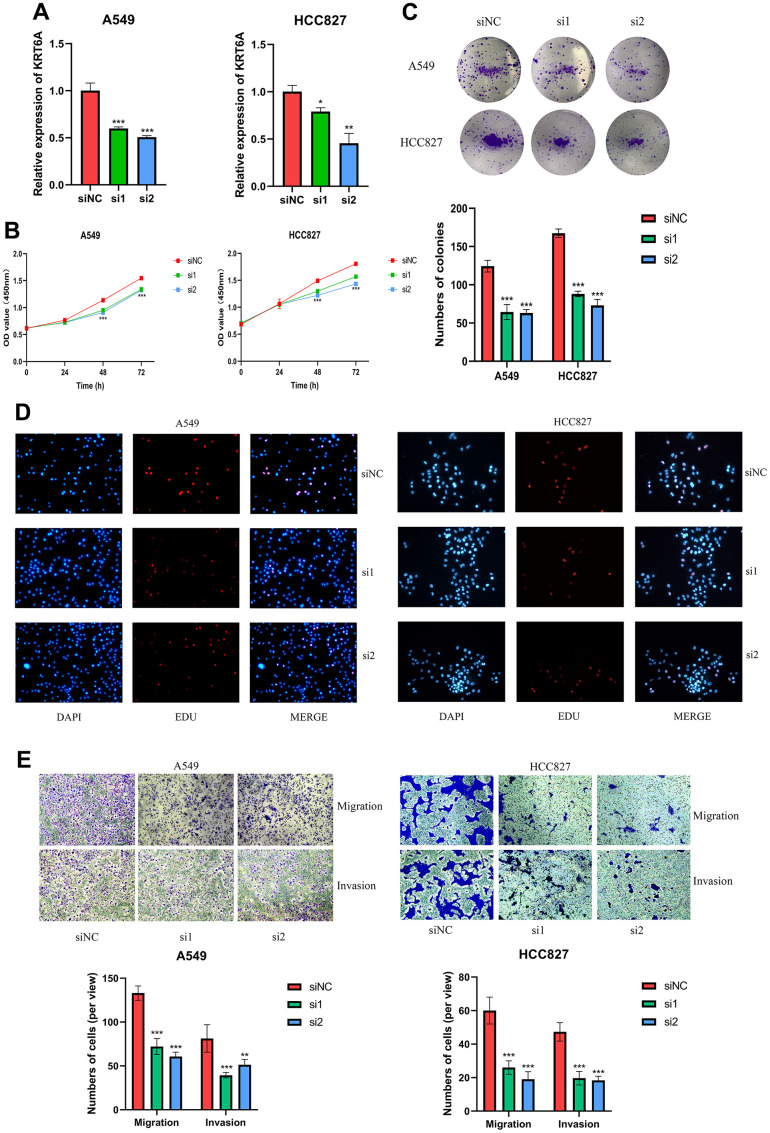
**KRT6A silencing inhibits LUAD cell proliferation, migration and invasion (A549 and HCC827).** (**A**) RT-qPCR was used to verify the efficiency of KRT6A knockdown in LUAD cell lines. (**B**) CCK-8, (**C**) clone formation and (**D**) EDU assays were used to test the proliferation of LUAD cell lines. (**E**) Transwell assay was applied to measure cell migration and invasion of LUAD cell lines. ns, not significant, *P < 0.05, **P < 0.01, ***P < 0.001, ****P < 0.0001.

Considering the important role of FA score in LUAD, the independent prognostic genes may have a greater impact on the biological function of LUAD cells. We chose KRT6A, exhibiting the most robust prognostic association, to further substantiate our conjecture. To explore the role of KRT6A in LUAD *in vitro*, siRNAs were introduced into A549 and HCC827 cells to suppress KRT6A expression, as evidenced by RT-qPCR (Figure 11A). The CCK-8 assay revealed that suppression of KRT6A led to a reduction in the total cell viability of both A549 and HCC827 cells (Figure 11B). The results of the clone formation and EDU assays corroborated the results of CCK8 (Figure 11C, 11D). In addition, a significant decrease in the LUAD cell invasion was also shown by the transwell assay (Figure 11E).

## DISCUSSION

Lipid metabolism disorder is an important metabolic change in cancer. Cancer cells can exploit a range of matrices and matrix sources to fulfill their catabolic and anabolic demands, including endogenous and exogenous FA, to maintain a high proliferation rate [34]. Dysregulation of fatty acid metabolism can promote proliferation, apoptosis, migration, and invasive capabilities of transformed cells, ultimately leading to tumorigenesis [35, 36]. Nonetheless, most preceding studies have predominantly concentrated on singular FARGs, while the comprehensive function of multiple FARGs in modulating one another remains insufficiently elucidated. Identifying the distinct fatty acid metabolic pattern in LUAD will contribute to comprehending tumorigenesis and cancer progression while offering valuable insights for the development of innovative LUAD therapeutics and prognostic approaches. The contemporary advancement of bioinformatics techniques facilitates potent, large-scale approaches for examining molecular markers and prognostic indicators across an array of malignancies and assorted maladies. In recent years, numerous multi-gene signatures have been successfully developed, allowing for precise prognostication of patient outcomes and therapeutic efficacy [37–39].

In this study, a comprehensive evaluation of the somatic mutations and mRNA expression of 38 FARGs in patients with TCGA-LUAD was performed, and 171 of 616 patients were observed to have mutations, while 28 FARGs were differentially expressed in cancer and adjacent tissues. Based on 38 FARGs, two different molecular subtypes (A and B) were identified. Subtype A patients had more advanced TNM stage and worse OS as compared to subtype B. The immune cell infiltration and enrichment pathways between both subtypes were analyzed and the results revealed that as compared to subtype B, the infiltration of most immune cells in subtype A was relatively low. Not surprisingly, patients with subtype A had a low OS rate due to suppression of the immune system. Astonishingly, the infiltration analysis of TME cells indicated that some patients with subtype A had relatively rich adaptive and innate immune cell infiltration, including NK cells, Treg cells and Th2 cells. Additionally, GSEA analysis revealed that the two FA subtypes were characterized by markedly distinct pathways. Subtype A was mainly concentrated in homologous recombination and DNA replication, while subtype B was concentrated in histidine metabolism and tyrosine metabolism.

Furthermore, 222 differentially expressed mRNAs were identified in different FA clusters in our study. The differentially expressed mRNA may possess regulatory associations with FARGs and contribute to tumorigenesis and progression, with 197 genes being correlated to the overall survival status of LUAD patients. Similar to the clustering results of FARGs typing, patients were divided into three gene clusters: A, B and C, according to the prognosis DEGs. The OS, FARGs expression and immune infiltration among gene clusters were different. Given the potential of FARGs to assess clinical outcomes and treatment responses, a scoring system named the FA score was developed. The FA score is based on five FARGs and has been validated across multiple cohorts for its predictive capability. These FARGs have been associated with tumorigenesis and some have been implicated in studies of LC. For example, overexpression of UBE2S was identified in human LC tissues and cell lines, and UBE2S knockout resulted in significant inhibition of the proliferation of lung cells and induced their apoptosis [40]. What is more, the UBE2S expression exhibited a negative correlation with the survival rate of patients with LUAD. The mechanism underlying the action of UBE2S involves its direct interaction with IκBα in LUAD to stimulate the NFκB pathway, which in turn activates the EMT signal to promote adenocarcinoma metastasis [41]. The overexpression of HMMR in LUAD predicts the poor prognosis of patients and is related to a variety of clinical indicators such as patient stage and smoking. The proliferation and migration of LUAD cells are promoted by the up-regulation of HMMR, which may be regulated by the TMPO-AS1/let-7b-5p axis [42]. Relevant studies have reported high expression of KRT6A in LC tissues, and the increased expression of KRT6A can be utilized to predict the prognosis of LUAD patients [43]. Here, we focused on the KRT6A, which shows the strongest prognostic correlation to the prognosis of LUAD patients. Functional experiments found that siRNA-induced knockdown of KRT6A inhibited LUAD cell proliferation, migration and invasion, suggesting that the oncogenic property of KRT6A in LUAD tumorigenesis and progression. However, this process needs further study to elucidate its detailed mechanisms. Survival analysis revealed that in comparison to the OS of the high FA score group, the OS of the low FA score group was prolonged, and similar results were obtained in most clinical subtypes. More importantly, we have proved that the FA score has a strong correlation with patients’ clinical symptoms and can be employed as an independent prognostic factor. Furthermore, nomograms were constructed utilizing the demographic features of age, sex, TNM staging, and FA score, thereby enabling clinicians to easily prognosticate patient outcomes. These results show that the FA score not only has a strong and reliable ability to predict the prognosis of patients but also consists of only a small number of genes, which is conducive to clinical translation.

TME is a complex network composed of immune cells, cytokines and fibroblasts, which plays a crucial role in cancer treatment and prognosis [44, 45]. Considering that TME and immune cell infiltration are related to cancer prognosis, it is necessary to explore the tumor immune microenvironment of LUAD. Our data revealed that people with high FA scores exhibit reduced immune and stromal cell infiltration and increased tumor purity. Furthermore, the augmentation index of the antineoplastic immunological cycle and the percentage of infiltrating immune cells in the group with diminished FA scores surpass those within the elevated FA score cohort, signifying a more robust inherent antineoplastic immunity within the tumor microenvironment of patients belonging to the group with reduced FA scores [46]. ICI has significantly improved the therapeutic prospects for patients with advanced NSCLC [47], and the response to ICI treatment can be predicted by the expression of an important biomarker, PD1/PD-L1 [48, 49]. However, PD1/PD-L1 seems not a perfect predictor and more research is being conducted to identify better predictors [50, 51]. Studies have shown that CTLA4, CD200 and CD80 are important immune checkpoints of LUAD. In this study, the FA score is associated with many immune checkpoints, including these three, which may be potential targets for the ICI treatment of LUAD patients. Methodically investigating the marker gene set between high and low FA score groups provides more information for us to deeply understand the transcriptome regulation mechanism of FA score in LUAD. It was found that the marker pathways with elevated levels in the high FA score group were related to recognized carcinogenic signaling pathways, including the MTORC1 pathway, Myc pathway, and cell cycle pathway [52]. The preliminary data strongly imply the internal relationship between immune-derived signals and carcinogenic pathways and this might help to develop new strategies for the discovery of candidate drugs in future research. The TIDE algorithm and IPS score, both have illustrated that the low FA score group responded better to immunotherapy. Moreover, after evaluating patients who received immunotherapy from IMvigor210 cohort, we observed that patients in the low FA score group displayed a higher proportion of response to immunotherapy, which once again verified the predictive value of the FA score. We also found a positive correlation between FA scores and various immunotherapy pathways which indicates that the FA score has the potential for immunotherapy guidance. TMB was identified as a biomarker of immunotherapy response, where the higher the TMB, the greater the benefits of immunotherapy [53, 54]. The patients with high FA scores were observed to have higher TMB, however, as mentioned above, patients in the high FA score group exhibited reduced immune activity, implying that a high value of TMB does not essentially indicate high immunogenicity. Detailed analysis revealed that due to mutations in 37 genes, high TMB score was observed in the high FA score group. Interestingly, the co-mutation frequency of these genes is very high, which indicates that the co-mutation of these genes may lead to unknown changes in TME regulation. Nonetheless, the effect of these co-mutations on patients’ response to immunotherapy requires further study. CNV is also an important factor affecting tumor immunity. In different cancers, a high level of CNV with TME has greater tumor-promoting and immunosuppressive characteristics [55]. Various studies have shown that high levels of CNV in LUAD cells result in the exponential proliferation of tumors and a decrease in immune infiltration. Specifically, focal CNV is primarily associated with increased tumor proliferation, while elevated levels of arm and whole chromosomal CNV predominantly correspond to decreased immune infiltration [56], which is consistent with the negative correlation between TMB and TME score in our results. Cells that evade anti-tumor monitoring may have high levels of CNV, caused by chromosomal instability. The results from our study showed that CNV and FGA increased simultaneously in the high FA score group, which was consistent with the observation that increased CNV results in an increased death rate [57].

When combined with radiotherapy, chemoradiotherapy and targeted drugs, immunotherapy has obvious synergistic effects [58]. In order to identify drugs that cooperate with patients’ immunotherapy and promote personalized treatment decisions, seven potential drugs were identified through the interaction analysis between FA score and drug response. Among the seven candidate drugs, leptomycin B is the first generation of chromosome region maintenance (CRM) inhibitor. The anti-cancer effect of leptomycin B has been detected in a variety of cancer cell lines, such as LC [59] and head and neck cancer [60]. In addition, leptomycin B combined with other drugs can enhance the sensitivity of cancer cells to chemotherapy drugs [61, 62]. Paclitaxel can bind and promote tubulin to assemble successfully dysfunctional microtubules. Microtubule dysfunction results in the inhibition of mitosis and cell proliferation, ultimately culminating in the death of rapidly dividing tumor cells. Combined with nano albumin and carboplatin, it can be used as the first-line treatment for patients with advanced NSCLC [63, 64]. Gemcitabine is a nucleoside antimetabolite that inhibits DNA synthesis [65] and this drug has shown promising results in phase I and early phase II studies of NSCLC [66]. Vincristine is an anti-mitotic cancer drug that blocks cancer cells in the metaphase and triggers their apoptosis [67]. Vincristine is rarely used alone for LC treatment; it is often combined with cyclophosphamide and doxorubicin as a second-line treatment for small cell lung cancer (SCLC) [68]. These studies also verify the reliability of chemotherapeutic drugs from our results.

Our study aims to classify LUAD patients into subtypes, identify DEGs and develop a prognosis model, and associate FARGs with patient prognosis. However, this study still has some limitations that need to be considered. The results of this study mainly come from bioinformatics analysis, although the results were verified in several independent public cohorts, prospective clinical studies are needed to confirm the clinical value of our FA score. Furthermore, due to the lack of mutation, CNV and other data in the GEO cohort, and multi-omics analysis can only be carried out in the TCGA cohort. Finally, our studies only preliminarily explored the functions of KRT6A in LUAD cells. To elucidate the molecular mechanisms underpinning the FA score’s effect on LUAD, more genetic modifications need to be undertaken to further confirm the role of the FA score on LUAD.

## CONCLUSIONS

In conclusion, this study first comprehensively describes the typing and prognostic value of FARGs in LUAD and constructs a FA score, which can be used to predict immune infiltration, ICIs response, drug vulnerability and prognosis in LUAD patients. Our findings suggest a novel target and prediction model for LUAD from the perspective of FA metabolism, as well as a possible association between chemotherapeutic sensitivity, metabolic reprogramming, and immune response activity.

## Supplementary Material

Supplementary Figures

Supplementary Table 1

Supplementary Tables 2-4

Supplementary Table 5

Supplementary Table 6

Supplementary Table 7

Supplementary Table 8

Supplementary Table 9

Supplementary Table 10

Supplementary Table 11

Supplementary Table 12

Supplementary Table 13

Supplementary Table 14

Supplementary Table 15

Supplementary Table 16

Supplementary Table 17

Supplementary Table 18

Supplementary Table 19

Supplementary Table 20

Supplementary Table 21

Supplementary Table 22
