# Supplementary material for: Identification of fatty acid-related subtypes, the establishment of a prognostic signature, and immune infiltration characteristics in lung adenocarcinoma
Source: Aging (Albany NY). 2023 May 16;15(10):4202–35. doi: 10.18632/aging.204725 (PMC10258028; doi:10.18632/aging.204725)
Supplement: Supplementary Figures [file aging-15-204725-s001.pdf]

SUPPLEMENTARY FIGURES

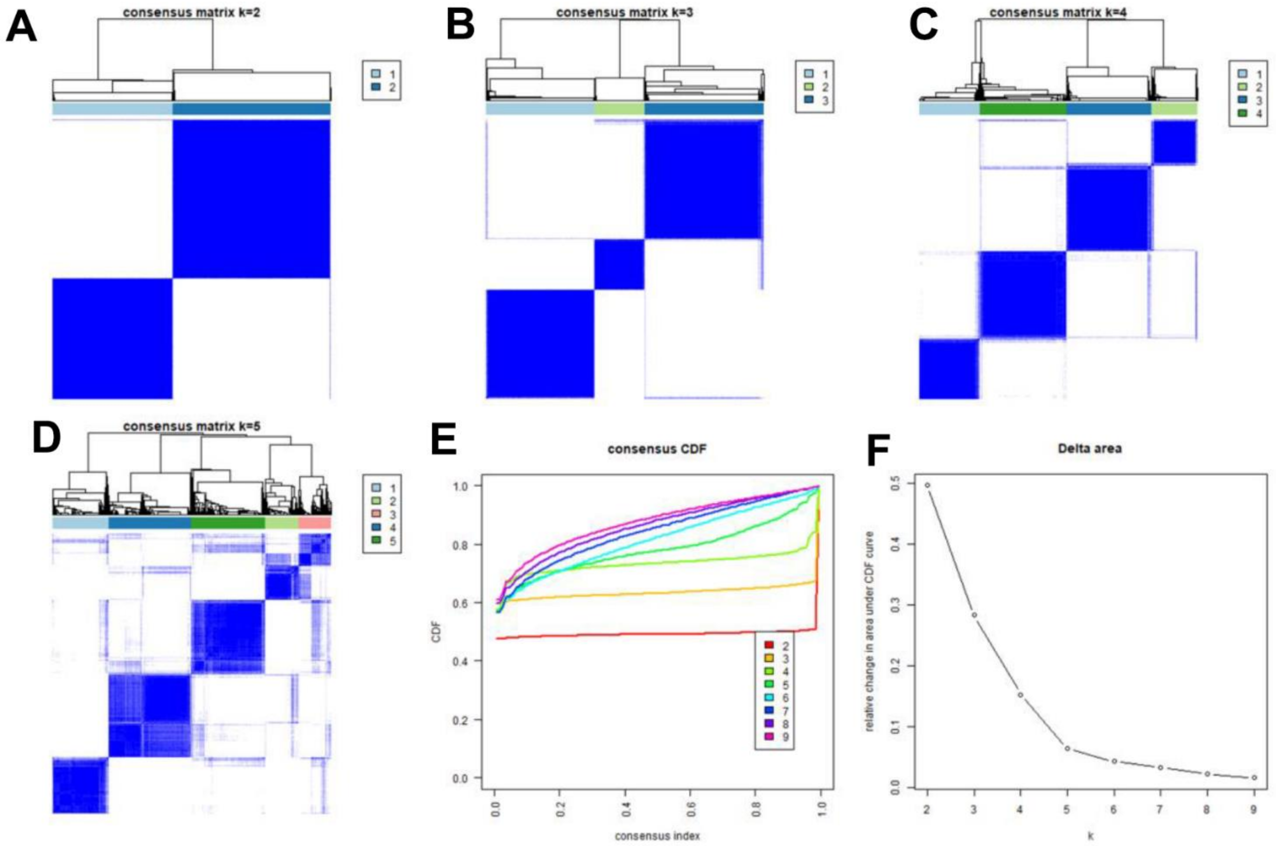

Supplementary Figure 1. Consensus matrixes of all LUAD patients based on FARGs. (**A**–**D**) Consensus matrixes of all LUAD patients for each k (k = 2–5). (**E**) Cumulative distribution function curves for unsupervised clustering of LUAD based on FARGs, k = 2-9. (**F**) Relative change in area under the CDF curve for unsupervised clustering of LUAD, k =2-9.

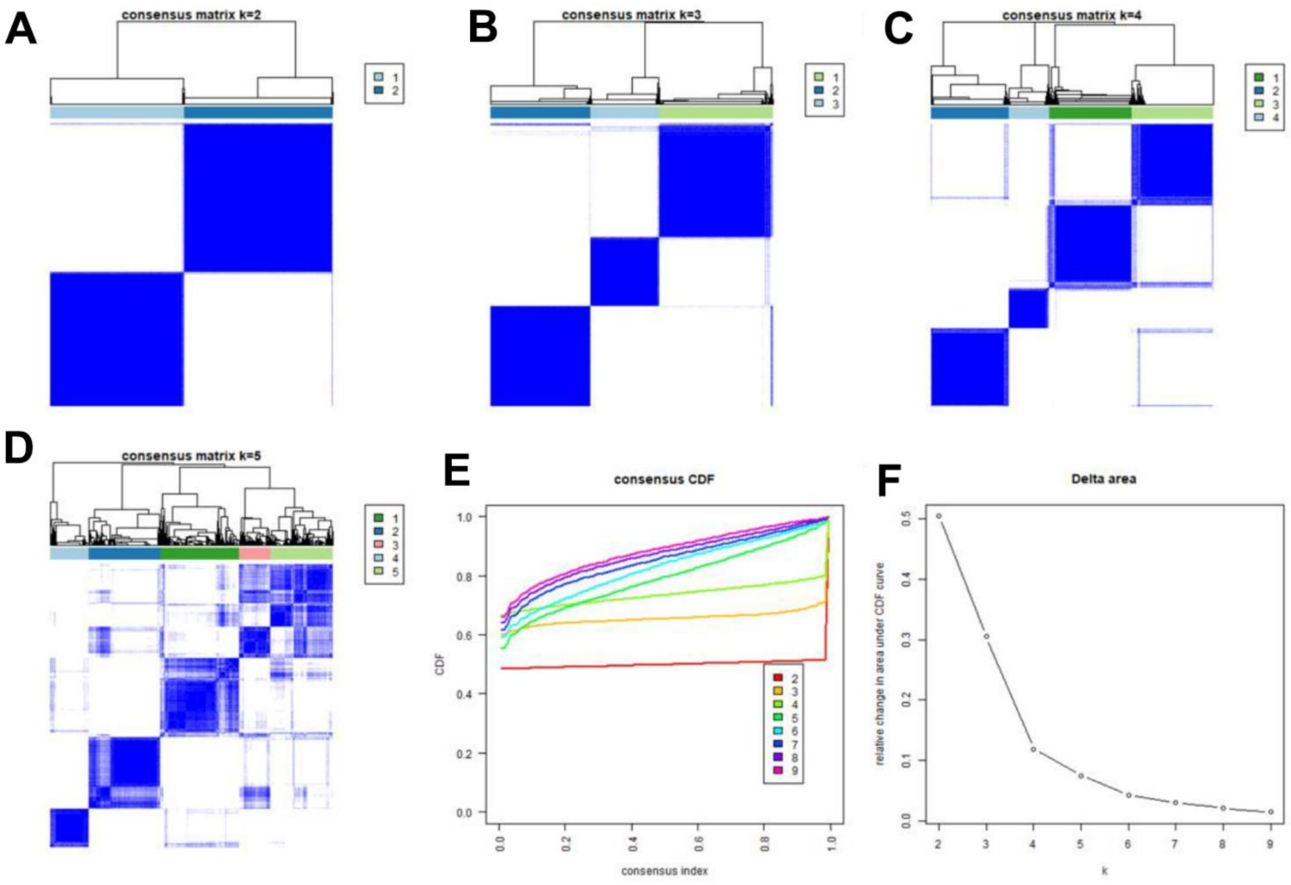

Supplementary Figure 2. Consensus matrixes of all LUAD patients based on prognostic DEGs. (**A**–**D**) Consensus matrixes of all LUAD patients for each k (k = 2–5). (**E**) Cumulative distribution function curves for unsupervised clustering of LUAD based on prognostic DEGs, k = 2-9. (**F**) Relative change in area under the CDF curve for unsupervised clustering of LUAD, k =2-9.

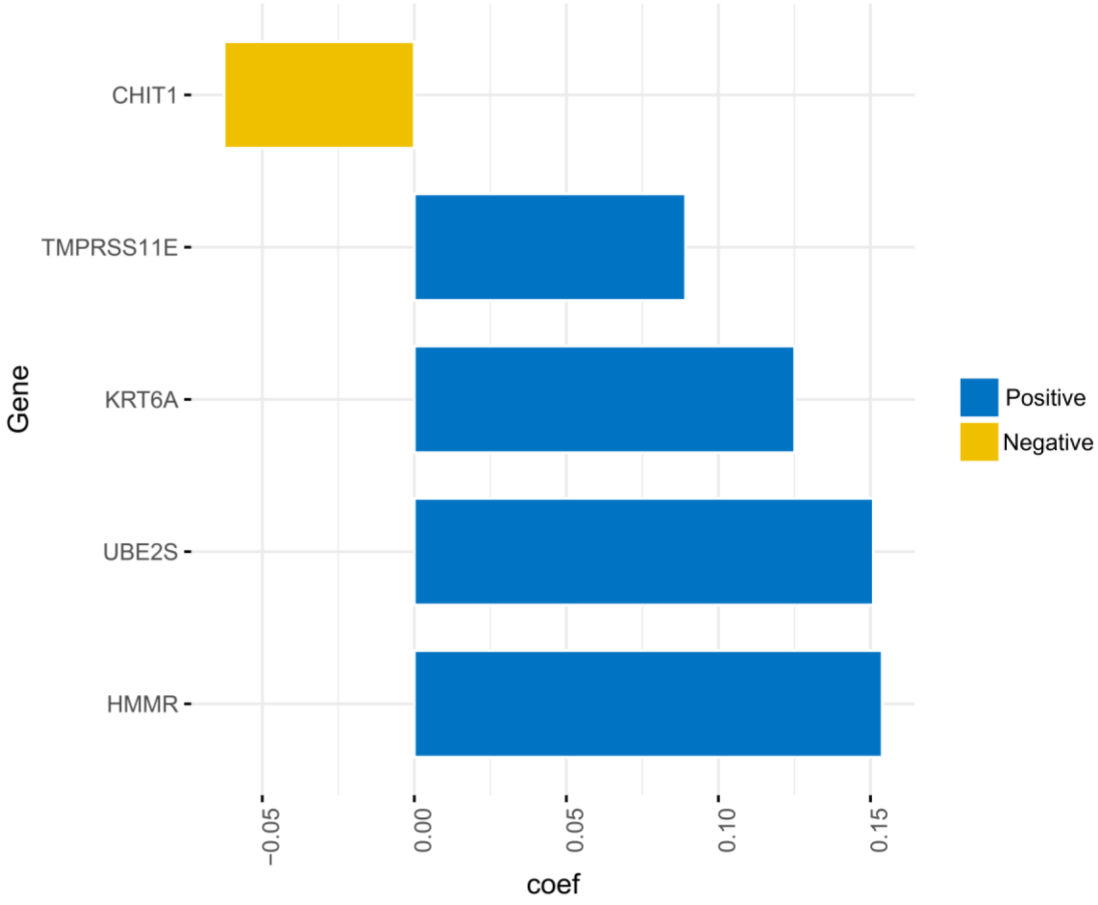

Supplementary Figure 3. Coefficient distribution of the genes in FA score.

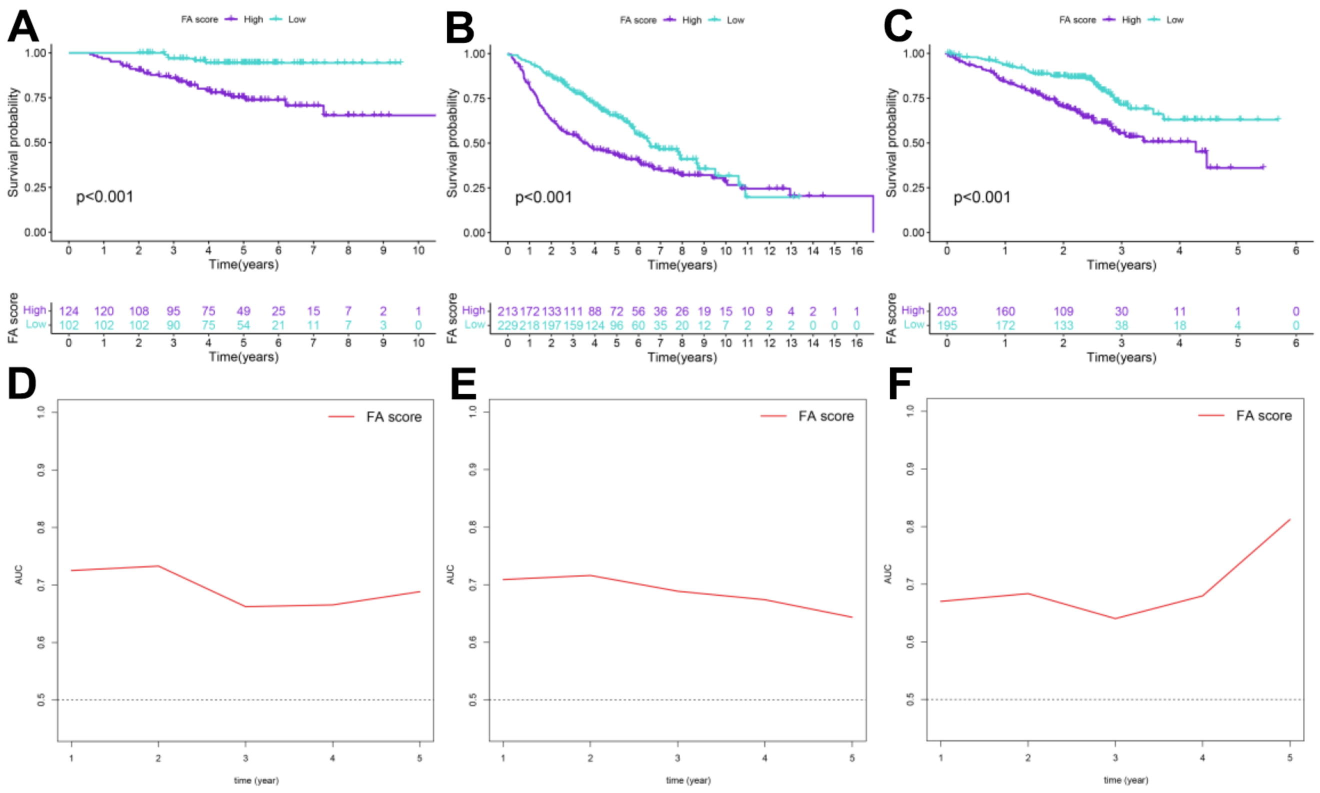

Supplementary Figure 4. Prognostic value of FA score in GEO cohorts. (**A**–**C**) Kaplan–Meier curves for patients with high and low FA score in the GSE31210, GSE68465 and GSE72094 cohorts. (**D**–**F**) Time-dependent receiver operating characteristic curve of FA score for predicting the prognosis of the LUAD patients in the GSE31210, GSE68465 and GSE72094 cohorts.

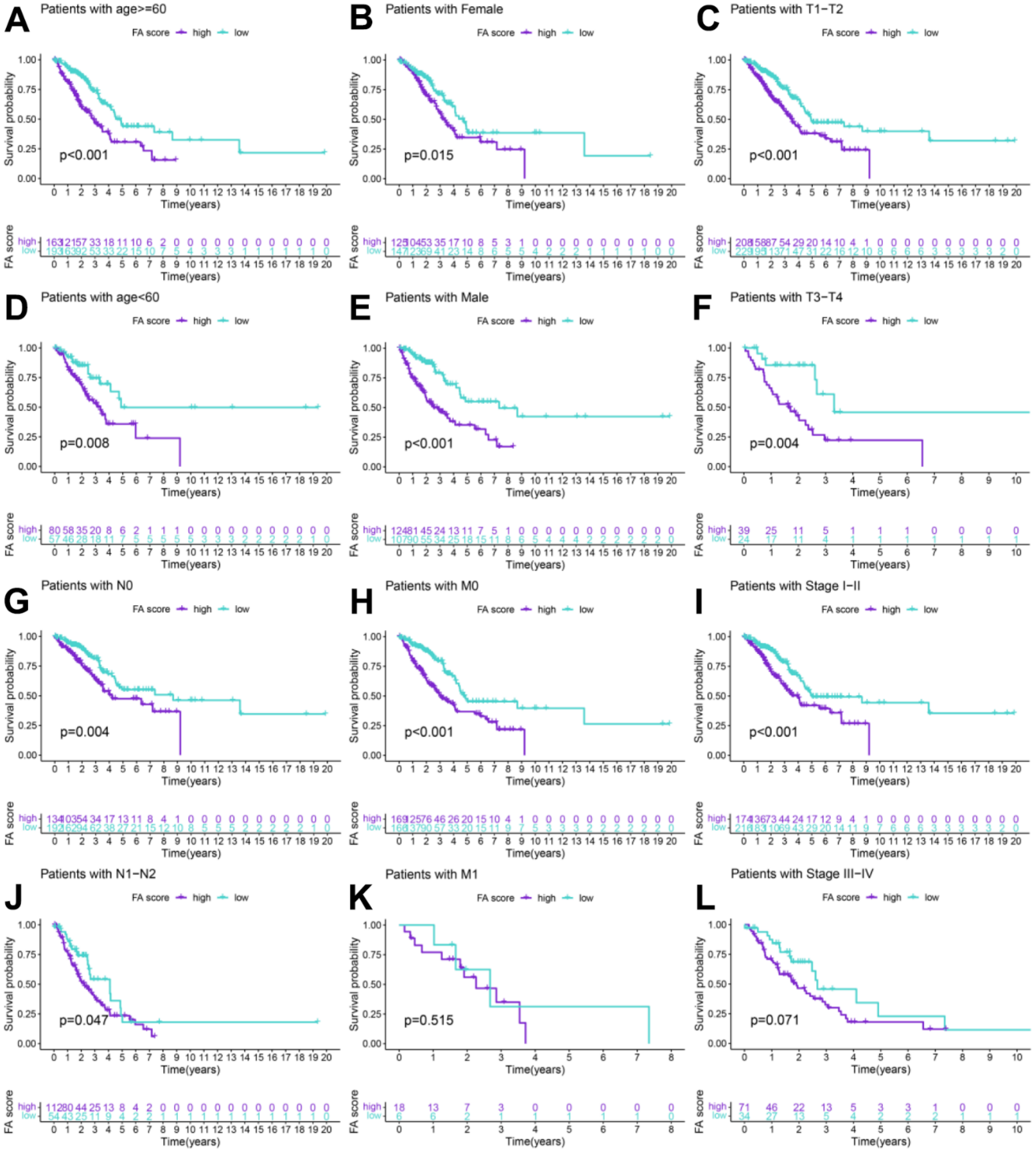

Supplementary Figure 5. The survival curves of the FA score stratified by age, gender, T, N, M and stage. (**A**) ≥60 years, (**B**) female, (**C**) T1-2, (**D**) <60 years, (**E**) male, (**F**) T3-4, (**G**) N0, (**H**) M0, (**I**) stage1-2, (**J**) N1-2, (**K**) M1, (**L**) stage3-4.

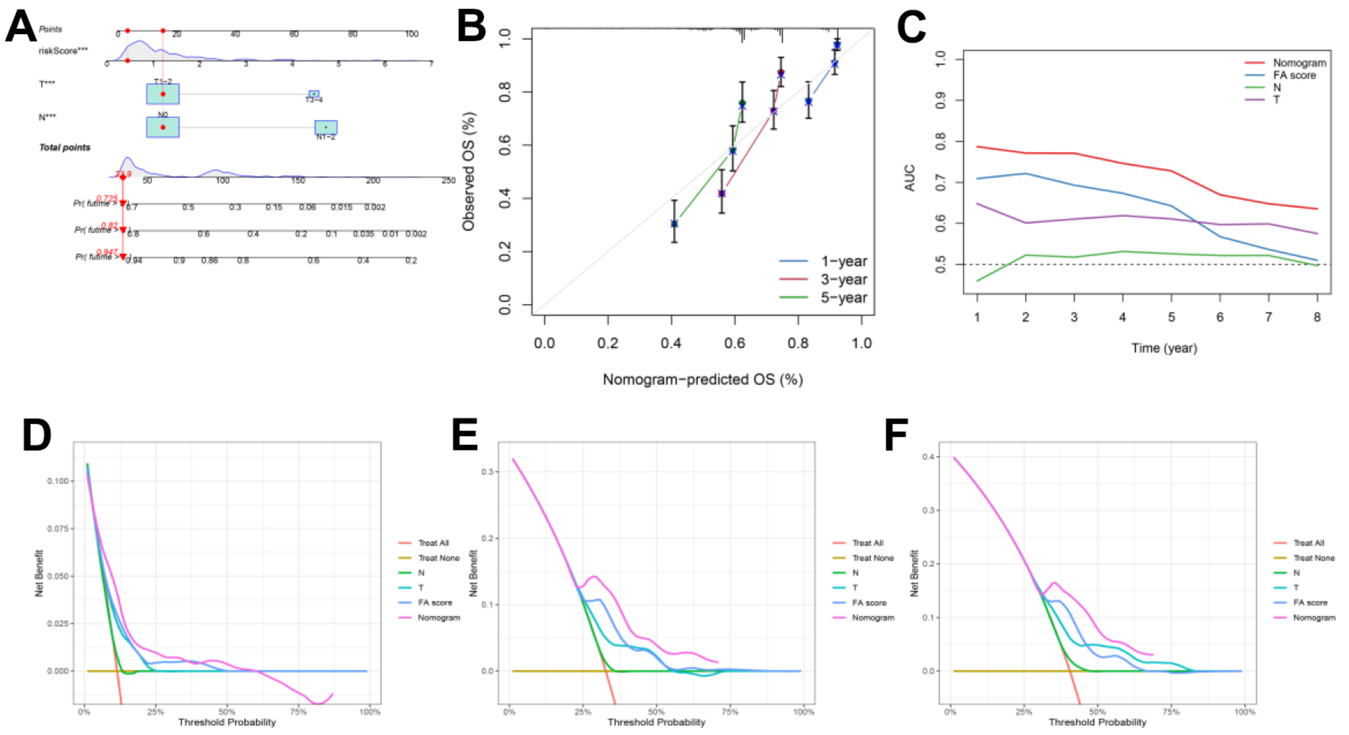

Supplementary Figure 6. Validation of a nomogram in the GSE68465 cohort. (**A**) Nomogram for predicting the 1-, 3-, 5-years OS. (**B**) Calibration curve of the nomogram for predicting the 1-, 3-, and 5-years OS. (**C**) ROC curve for predicting the different years' OS. (**D**–**F**) Decision curves showing the comparison of net benefits of the nomogram, N, T and FA score for 1-, 3-, and 5-years OS. ns, not significant, *P < 0.05, **P < 0.01, ***P < 0.001, ****P < 0.0001.

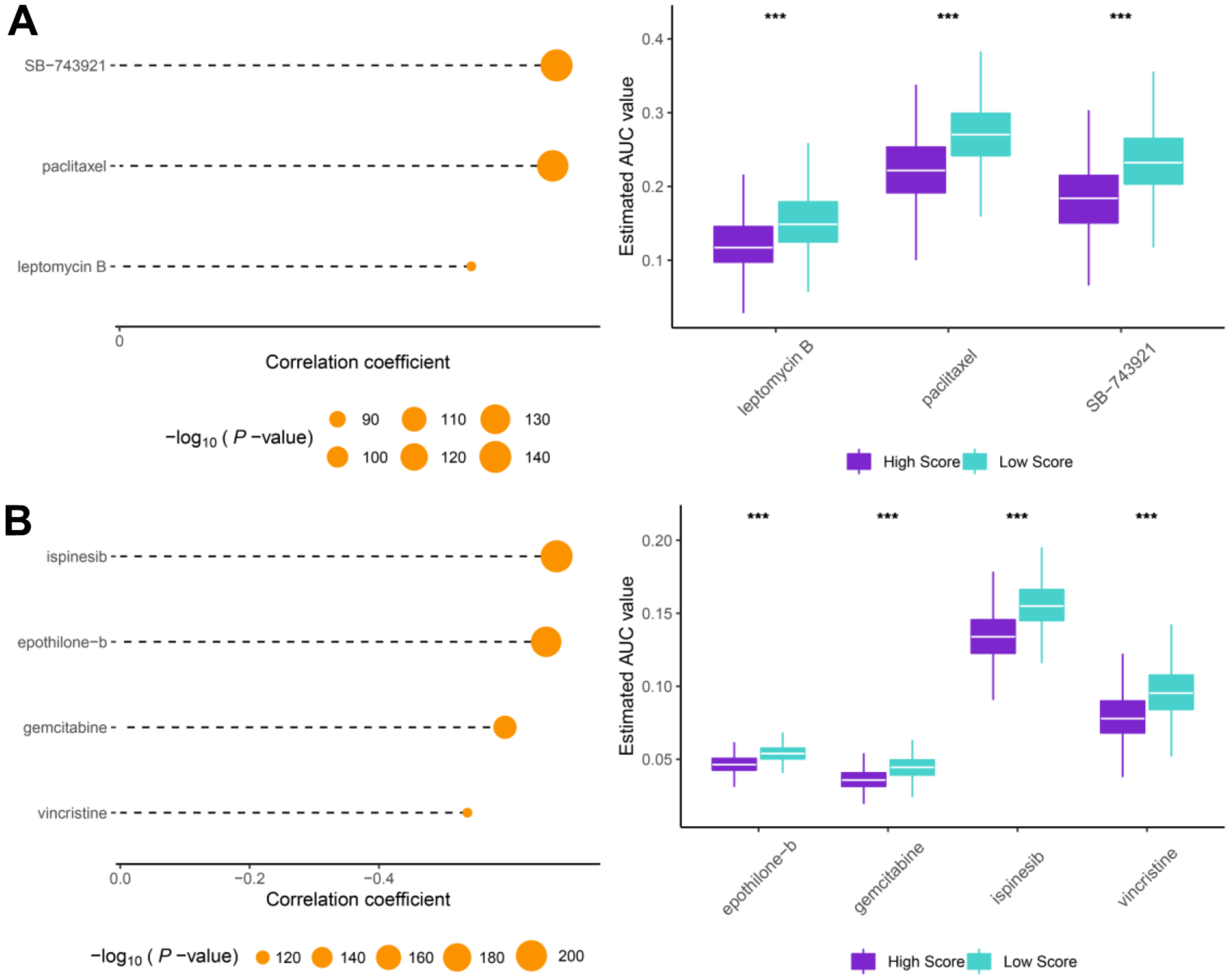

Supplementary Figure 7. Prediction of the sensitivity to chemotherapy drugs targeting the FA score. (**A**) Three CTRP-related compounds were identified by Spearman correlation analysis between the FA score and AUC value. (**B**) Four PRISM-related compounds were identified by Spearman correlation analysis between the FA score and AUC value. ns, not significant, *P < 0.05, **P < 0.01, ***P < 0.001, ****P < 0.0001.

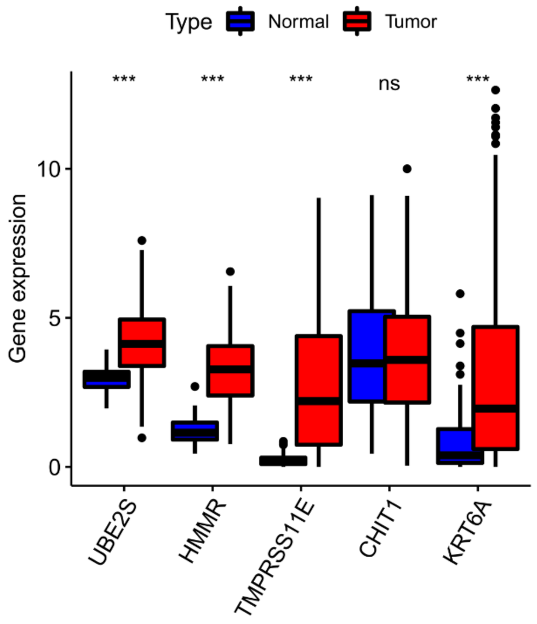

Supplementary Figure 8. The expression exploration of the genes in the FA score based on the TCGA cohort.
