# Supplementary material for: Identification of fatty acid-related subtypes, the establishment of a prognostic signature, and immune infiltration characteristics in lung adenocarcinoma
Source: Aging (Albany NY). 2023 May 16;15(10):4202–35. doi: 10.18632/aging.204725 (PMC10258028; doi:10.18632/aging.204725)
Supplement: Supplementary Tables 2-4 [file aging-15-204725-s003.pdf]

Supplementary Table 2. The name list of fatty acid genes.

CPT1A
ACADS
ALDH1B1
ACADSB
ACADL
ALDH2
ACADM
CYP4A11
ACAT2
ACADVL
ACAT1
ACAA2
HADH
HADHB
HADHA
ADH7
ADH6
ACSL6
ADH1B
ADH1C
ECHS1
ADH5
ALDH9A1
ALDH3A2
ACSL5
ADH1A
EHHADH
GCDH
ALDH7A1
ACOX3
ACSL1
ACAA1
CPT2
ACOX1
ECI2
ECI1
ACSL3
ACSL4

Supplementary Table 3. The primer sequences of the genes.

Primers	Primers sequence (5′-3′)	
**Gene names**	**Forward primer**	**Reverse primer**
KRT6A	GCGTTGGAGGTGGCTTCAGTTC	AGGAGGTGGTGGTGTACTTGATGG
CHIT1	CCTCAACGTGGATGCTGCTGTG	TGTGAAGGAGCGTCCGTAGGTAG
TMPRSS11E	ACCTCGACCTTCACAGGACTCTTC	CACTGCCAGGACAATCAGGGATATG
UBE2S	TGCCTGCTGATCCACCCTAACC	AGCCGCATACTCCTCGTAGTTCTC
HMMR	AACAAGTGGCGTCTCCTCTATGAAG	TGTTCCTGAGCTGCACCATGTTC
GAPDH	CCAGCAAGAGCACAAGAGGAAGAG	GGTCTACATGGCAACTGTGAGGAG

Supplementary Table 4.The sequences of the siRNA.

	siRNA sequence	
**Names**	**Forward sequence**	**Reverse sequence**
siRNA NC	UUCUCCGAACGUGUCACGU TT	ACGUGACACGUUCGGAGAA TT
siRNA 1	CCUACAUGAACAAGGUUGA TT	UCAACCUUGUUCAUGUAGG TT
siRNA 2	GCGUUGGACAAGUCAACAU TT	AUGUUGACUUGUCCAACGC TT
